# *APOER2* splicing repertoire in Alzheimer’s disease: Insights from long-read RNA sequencing

**DOI:** 10.1371/journal.pgen.1011348

**Published:** 2024-07-22

**Authors:** Christina M. Gallo, Sabrina A. Kistler, Anna Natrakul, Adam T. Labadorf, Uwe Beffert, Angela Ho

**Affiliations:** 1 Department of Biology, Boston University, Boston, Massachusetts, United States of America; 2 Department of Pharmacology, Physiology & Biophysics, Boston University Chobanian & Avedisian School of Medicine, Boston, Massachusetts, United States of America; 3 Bioinformatics Program, Boston University, Boston, Massachusetts, United States of America; 4 Department of Neurology, Boston University Chobanian & Avedisian School of Medicine, Boston, Massachusetts, United States of America; University of Miami Miller School of Medicine, UNITED STATES OF AMERICA

## Abstract

Disrupted alternative splicing plays a determinative role in neurological diseases, either as a direct cause or as a driver in disease susceptibility. Transcriptomic profiling of aged human postmortem brain samples has uncovered hundreds of aberrant mRNA splicing events in Alzheimer’s disease (AD) brains, associating dysregulated RNA splicing with disease. We previously identified a complex array of alternative splicing combinations across *apolipoprotein E receptor 2* (*APOER2*), a transmembrane receptor that interacts with both the neuroprotective ligand Reelin and the AD-associated risk factor, APOE. Many of the human *APOER2* isoforms, predominantly featuring cassette splicing events within functionally important domains, are critical for the receptor’s function and ligand interaction. However, a comprehensive repertoire and the functional implications of *APOER2* isoforms under both physiological and AD conditions are not fully understood. Here, we present an in-depth analysis of the splicing landscape of human *APOER2* isoforms in normal and AD states. Using single-molecule, long-read sequencing, we profiled the entire *APOER2* transcript from the parietal cortex and hippocampus of Braak stage IV AD brain tissues along with age-matched controls and investigated several functional properties of *APOER2* isoforms. Our findings reveal diverse patterns of cassette exon skipping for *APOER2* isoforms, with some showing region-specific expression and others unique to AD-affected brains. Notably, exon 15 of *APOER2*, which encodes the glycosylation domain, showed less inclusion in AD compared to control in the parietal cortex of females with an *APOE* ɛ3/ɛ3 genotype. Also, some of these *APOER2* isoforms demonstrated changes in cell surface expression, APOE-mediated receptor processing, and synaptic number. These variations are likely critical in inducing synaptic alterations and may contribute to the neuronal dysfunction underlying AD pathogenesis.

## Author summary

Alternative splicing enhances genetic diversity by producing various RNA isoforms from a single gene. The gene *LRP8*, which encodes apolipoprotein E receptor 2 (APOER2), undergoes extensive alternative splicing in the brain. APOER2 is a type I transmembrane receptor that interacts with extracellular ligands, notably the neuroprotective Reelin and APOE, a risk factor associated with Alzheimer’s disease (AD). Interestingly, alternative splicing of *APOER2* has been shown to be altered in AD; yet the complete repertoire of *APOER2* isoforms in both normal and AD-affected brains remained largely unexplored. Our study offers insight into the diverse isoforms of *APOER2* in the human brain and their splicing modifications in the context of AD. We discovered a range of combinatorial *APOER2* alternative splicing events, leading to a diverse set of isoforms in the human brain. Our analysis revealed both region- and disease-specific *APOER2* isoforms, indicating that *APOER2* splicing is not only spatially regulated but also undergoes alterations in AD. Moreover, we observed that some of the *APOER2* isoforms in AD brains show changes in cell surface expression, receptor processing and synapse numbers. These findings suggest that combinatorial splicing of *APOER2* is a critical determinant of its protein function and undergoes significant alterations in AD.

## Introduction

Over 90% of human genes undergo alternative splicing to encode proteins with different functions [1]. It is estimated that 10–15% of disease-causing mutations are located at splice sites [2,3]. This number increases when considering mutations that impact splicing regulatory elements such as splicing silencers and enhancers. Notably, the brain exhibits a higher expression of alternatively spliced genes compared to other tissues, particularly within neurons [4,5]. Hence, disruptive splicing plays a significant role in neurological diseases, either as a direct causative factor, or as a contributor to disease susceptibility [6]. In support of this, comprehensive gene expression studies that compared the transcriptomes of cognitively normal individuals with those affected by Alzheimer’s disease (AD) and younger brains revealed hundreds of unique RNA splicing alterations specific to AD and aging [7–9].

We previously identified a vast array of alternative splicing combinations within apolipoprotein E receptor 2 (*APOER2*) in the vertebrate brain [10]. APOER2, a transmembrane receptor, is known for binding the neuroprotective ligand Reelin and AD related risk factor, APOE. *APOER2* is particularly prone to cassette exon splicing events, where entire exons are selectively included or excluded from pre-mRNAs. This splicing variability allows for the addition or removal of key functional domains, impacting APOER2’s interaction with ligands and downstream signaling function [10,11]. Notably, mouse *Apoer2* ranks among the top ten genes exhibiting neuron-specific splicing events [12]. In the context of AD, alternative splicing of *APOER2* has been differentially observed, with lower inclusion rates of exon 18 (ex18 in humans, ex19 in mice) compared to individuals without cognitive impairment [13]. Interestingly, human *APOER2* ex18 inclusion has been positively correlated with global cognition [13], indicating potential isoform-specific roles of *APOER2* in cognitive processes. For instance, the exclusion of *Apoer2* ex19 in mice disrupts Reelin-induced hippocampal long-term potentiation (LTP), underscoring the importance of *Apoer2* splicing in mediating Reelin-induced synaptic plasticity [14]. Furthermore, enhancing *Apoer2* ex19 inclusion in an AD mouse model was found to ameliorate spatial learning deficits [13], suggesting that modulating *Apoer2* splicing could benefit learning functions.

In humans, a variety of alternative splicing events within *APOER2* in the brain have been documented [15–17]. RNA sequencing (RNAseq) studies on human brain tissue such as Genotype-Tissue Expression (GTEx) Portal, have further extended the diversity of *APOER2* splicing events. Utilizing single molecule, long-read RNAseq, we identified numerous diverse and novel human *APOER2* isoforms in the cerebral cortex giving rise to an extensive range of splicing combinations throughout the *APOER2* transcript, indicating possible differential functional impacts at the protein level [10,15]. However, it remains unclear whether the splicing landscape of *APOER2* shifts in AD brains.

In this study, we conducted a comprehensive profiling of the entire human *APOER2* transcript from the parietal cortex and hippocampus of Braak stage IV AD brain tissues using age-matched controls for comparison using single molecule, long-read RNAseq. We identified over 200 unique *APOER2* isoforms in the parietal cortex and hippocampus, with 151 isoforms in common to both, and several *APOER2* variants unique to AD. We found dysregulation in *APOER2* at both individual exon and full-length transcript levels in AD-affected brain regions. In addition, *APOER2* isoforms in AD demonstrated changes in cell surface expression and APOE-mediated receptor processing, indicating combinatorial splicing across *APOER2* could play a key role in dictating neuronal function in AD.

## Results

### *APOER2* transcript mapping in the human AD parietal cortex

**Fig 1:**
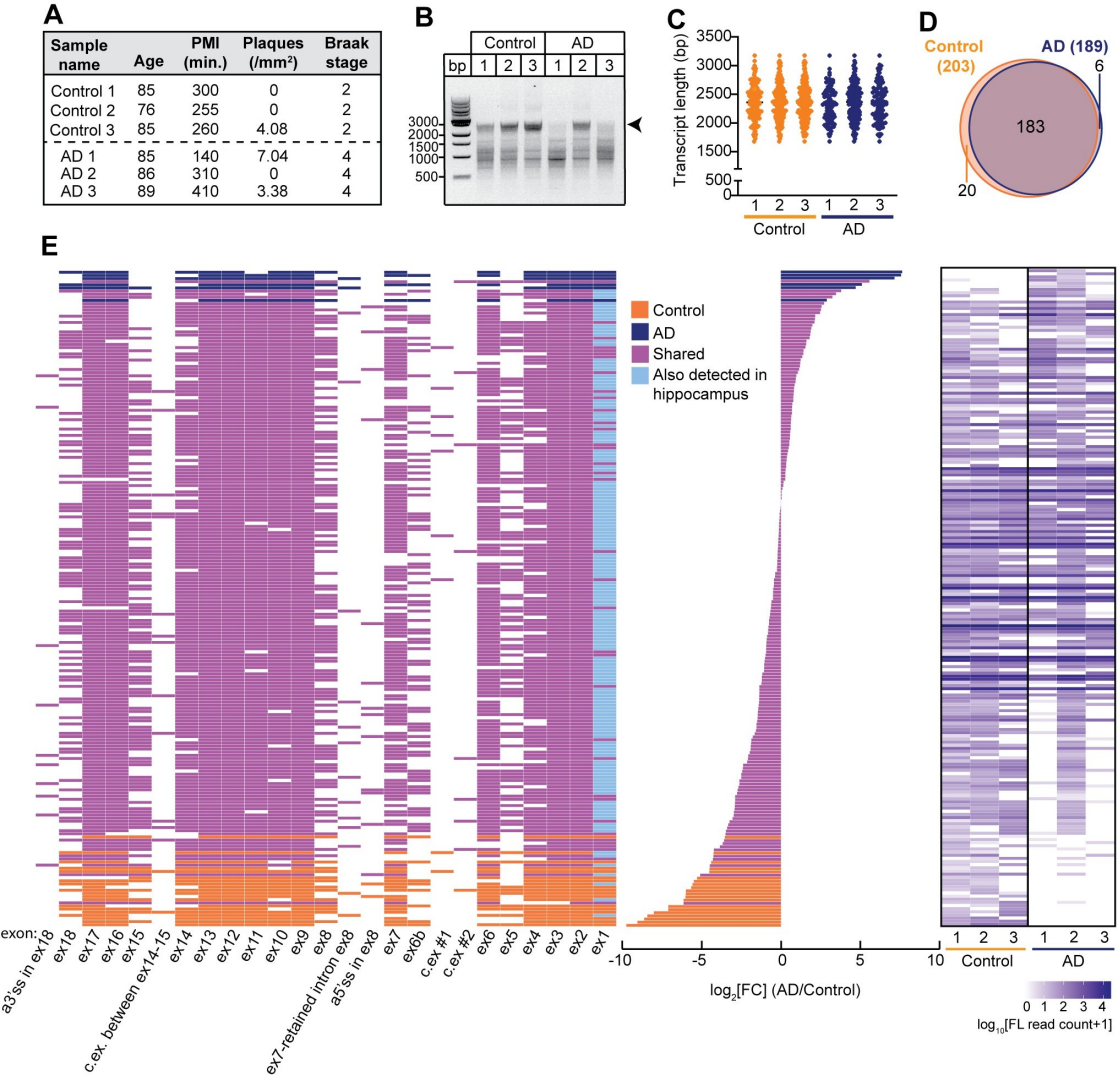
*APOER2* exhibits isoform diversity in the human parietal cortex. (A) Post-mortem parietal cortex tissue sample characteristics that includes age, postmortem interval (PMI), plaques per mm^2^ and Braak stage. (B) DNA gel depicting *APOER2* specific cDNA amplicons. Arrowhead indicates the expected size of full-length *APOER2* transcripts. (C) Graph depicting the transcript length distribution and mean of detected isoforms in the parietal cortex in base pairs (bp). Length does not include the RT-PCR primer sequences. (D) Venn diagram of detected *APOER2* isoforms in control and AD parietal cortex samples. (E) 209 unique *APOER2* transcripts detected in the human parietal cortex. Left: Transcript matrix depicting 209 individual *APOER2* isoforms as individual rows and exons as columns. Colored boxes indicate exon inclusion, while white boxes indicate exon exclusion. Isoforms are color coded based on whether they are common and shared to both control and AD (purple), specific to control (orange), or AD (navy blue). Transcripts that were also identified in the hippocampus (Fig 2) are indicated by a light blue coloring of constitutive exon 1. Middle: Bar plot indicating the log_2_FC (AD/control) of each corresponding transcript in the adjacent matrix. Right: Heat map indicating the log_10_ transformed number full-length (FL) reads per isoform for each sample.

To map the *APOER2* isoform landscape in human postmortem AD brains, we isolated total RNA from the parietal cortex of three individuals with Braak stage IV pathology, and three non-AD age-matched controls (**Fig 1A**). All individuals were female and had an *APOE* ɛ3/ɛ3 genotype. RNA was subjected to an *APOER2* specific cDNA synthesis, and RT-PCR was used to amplify the entire *APOER2* coding region (**Fig 1B**). cDNA amplicons underwent library preparation followed by single molecule, long-read RNAseq and were analyzed with PacBio’s IsoSeq analytic pipeline followed by a custom bioinformatic analysis to attain high-confidence *APOER2* isoforms. All samples returned over 70% of their full-length reads as *APOER2* isoforms, and the remaining off target read sequences were filtered out (**S1 Table**).

To examine the *APOER2* isoform pool, we analyzed the length in base pairs (bp) of the identified *APOER2* isoforms and found the mean length across all six samples clustered just under 2500 bp (**Fig 1C**). Since the expected full-length coding sequence of *APOER2* based on the RT-PCR primer scheme is 2892 bp, this suggested the presence of alternative splicing events within the identified *APOER2* transcripts. To determine how many detected *APOER2* isoforms make up the majority of *APOER2* full-length reads, we calculated the cumulative proportion of each isoform within each sample and found about 6–9 isoforms make up 60% of total *APOER2* reads within a given sample (**S1A Fig**). We next compared the number of *APOER2* isoforms detected between control and AD samples and found 183 *APOER2* isoforms in common between control and AD samples. However, there were 20 and 6 *APOER2* isoforms unique to control and AD, respectively in the parietal cortex for a total of 209 *APOER2* isoforms (**Fig 1D** and **S2 Table**).

In the parietal cortex, individual *APOER2* isoforms exhibit a plethora of alternative splicing events with cassette exon skipping or inclusion (**Fig 1E**). *APOER2* ex19, which encodes the last 12 amino acids of APOER2 and the 3’-untranslated regions, is not labeled as an individual exon, since the primer placement was at the nucleotides encoding the stop codon of the protein, capturing only a small segment of ex19. Twenty-five exons were identified across the 209 *APOER2* isoforms in the parietal cortex, including the canonical *APOER2* exons, as well as three cassette exons (two between ex6 and ex6B, and one between ex14 and ex15), an intron retention event between ex7 and ex8, and the usage of alternative splice sites in ex8 and ex18 (**S3 Table**).

To determine whether these exons were novel or have been previously annotated, we compared the identified exons to those annotated in Ensembl [release 105, geneID: ENSG00000157193.18], and only found the alternative splice site in ex18, and the cassette exon between ex14-15 previously annotated. The alternative splice site identified in ex8, and the two cassette exons between ex6-6B appear to be novel. We also examined the exon coordinates of those exons annotated for *APOER2* in the GTEx Project (v8.0, accessed 2022-01-14) and found similar results. However, there was an exon present in the GTEx database between ex6-6B that shared a 3’splice site with the two cassette exons we identified (**S4 Table**). As such, this may be an alternative exon with numerous 5’splice site choices.

### *APOER2* transcript mapping in the human AD hippocampus

**Fig 2:**
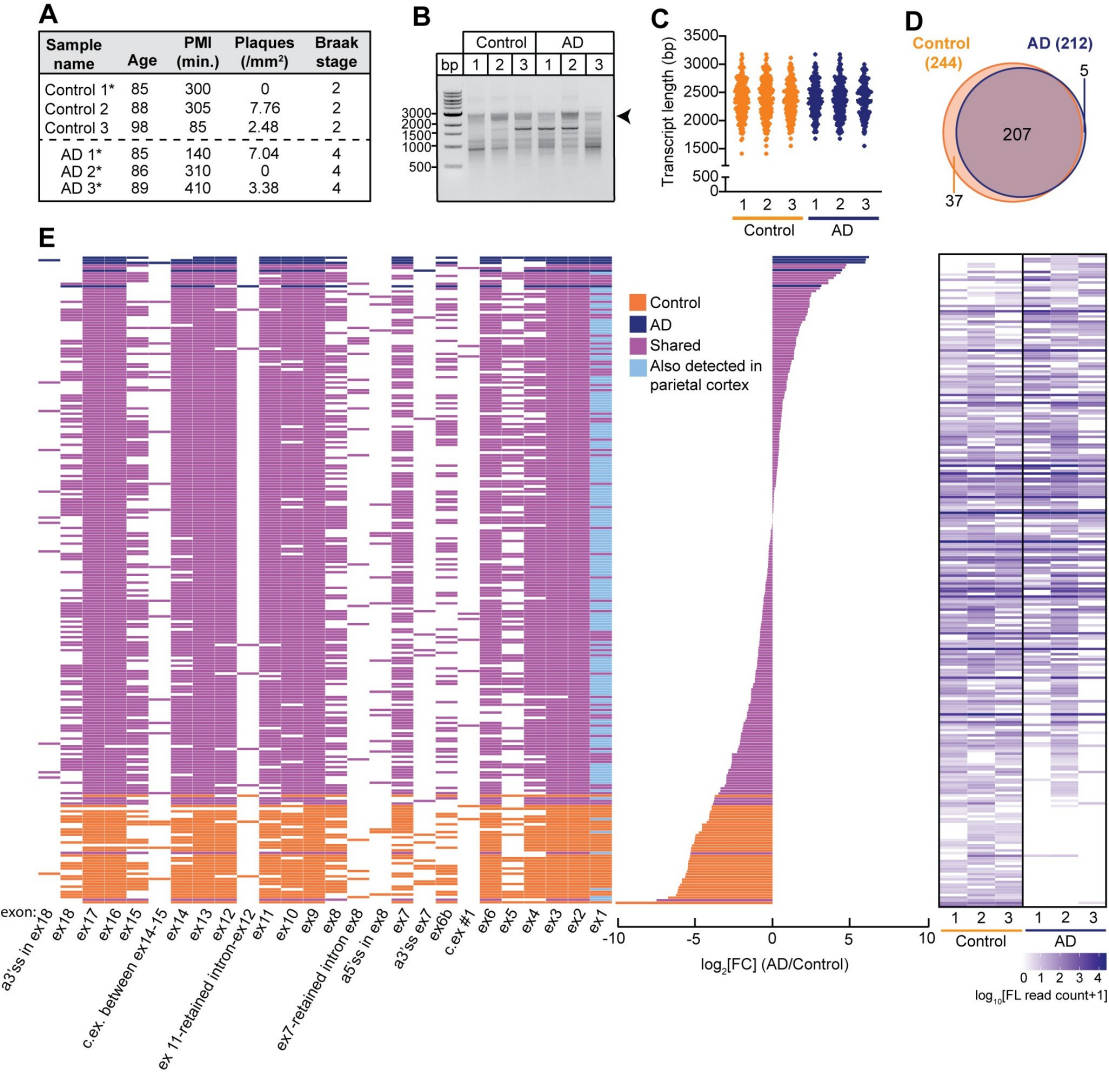
*APOER2* exhibits isoform diversity in the human hippocampus. (A) Postmortem hippocampal tissue sample characteristics that includes age, postmortem interval (PMI), plaques per mm^2^ and Braak stage. Asterisks indicate samples common between the parietal cortex and hippocampus. (B) DNA gel depicting *APOER2* specific cDNA amplicons. Arrowhead indicates the expected size of full-length *APOER2* transcripts. (C) Graph depicting the transcript length distribution and mean of detected isoforms in the hippocampus in base pairs (bp). (D) Venn diagram of detected isoforms in control and AD hippocampal samples. (E) 249 unique *APOER2* transcripts detected in the human hippocampus. Left: Transcript matrix depicting 249 individual *APOER2* isoforms as individual rows and exons as columns. Colored boxes indicate exon inclusion, while white boxes indicate exon exclusion. Isoforms are color coded based on whether they are common to both control and AD (purple), specific to control (orange), or AD (navy blue). Transcripts that were also identified in the parietal cortex (Fig 1) are indicated by a light blue coloring of constitutive exon 1. Middle: Bar plot indicating the log_2_FC (AD/Control) of each corresponding transcript in the adjacent matrix. Right: Heat map indicating the log_10_ transformed number of full-length (FL) reads per isoform for each sample.

To understand how *APOER2* splicing changes across AD relevant brain regions, we generated *APOER2*-specific long-read sequencing data of hippocampal tissue from the same 3 AD patients (indicated by asterisks), and 3 age-matched controls of which one was obtained from the same individual as the parietal cortex (**Fig 2A**), and subjected them to the same bioinformatic pipeline as the parietal cortex samples. The mean transcript length was clustered just under 2500 bp, and samples demonstrated comparable cumulative isoform frequencies, with 6–7 transcripts making up about 60% of full-length *APOER2* reads (**Figs 2B**, **2C,** and **S1B**). In the hippocampus, we identified 207 shared isoforms between control and AD samples, as well as 37 and 5 isoforms unique to control and AD, respectively (**Fig 2D** and **S5 Table**).

In total, 249 unique *APOER2* isoforms were identified in the human hippocampus (**Fig 2E**) with many exon splicing events, as was observed in the parietal cortex. In addition to the canonical *APOER2* full-length exons, we observed two of the cassette exons we identified in the parietal cortex, the same alternative splice sites in ex8 and ex18, and retention of the intron between ex7 and ex8 (**S3 Table**). Also, we found retention of the intron between ex11 and ex12, and use of an alternative 5’splice site before ex7. We did not observe one of the cassette exons that was identified in the parietal cortex (c.ex.#2), which is a shorter version of the other cassette exon (c.ex.#1) using a different 5’ splice site. We compared the full-length isoforms detected in the hippocampus to those detected in the parietal cortex and found 151 transcripts in common between the two regions (indicated by light blue boxes in ex1 of **Figs 1E** and **2E**).

### Top *APOER2* transcripts expressed in parietal cortex and hippocampus

**Fig 3:**
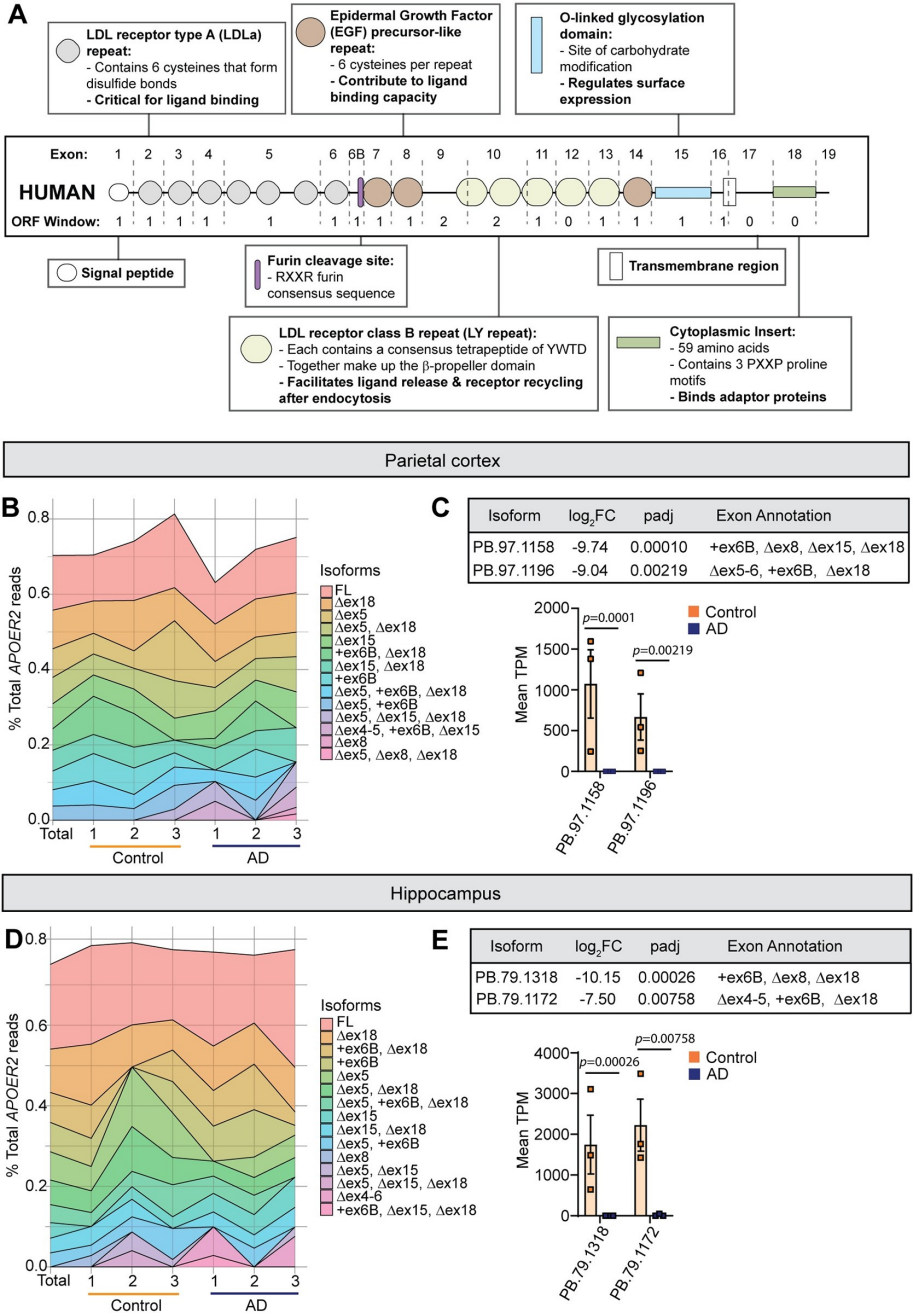
Top 10 expressed *APOER2* isoforms in the parietal cortex and hippocampus. (A) Human *APOER2* exon structure and protein functional domains. (B) Stacked area chart depicting the percent of full-length *APOER2* reads each of the top 10 isoforms per individual sample or across all samples (Total) make up in the parietal cortex. (C) Table showing adjusted *p*-value and exon annotation of *APOER2* transcripts. Bar plot of the mean *APOER2* TPM per group ± S.E.M. between control and AD. Statistical significance was calculated using DeSeq2, with an FDR of 0.1. (D) Stacked area chart depicting the percent of full-length *APOER2* reads each of the top 10 isoforms per individual sample or across all samples (Total) make up in the hippocampus. (E) Table showing adjusted *p*-value and exon annotation of *APOER2* transcripts. Bar plot of the mean *APOER2* TPM per group ± S.E.M. between AD and control. Statistical significance was calculated using DeSeq2, with an FDR of 0.1.

We next examined the top 10 expressed isoforms in each of the six parietal cortex samples, and across all the samples. The most abundant *APOER2* isoforms are largely consistent across individuals, regardless of AD status. The canonical full-length (FL) *APOER2* was the most abundant isoform identified, closely followed by *APOER2* lacking ex18 (Δex18), which encodes the cytoplasmic insert of the receptor (**Fig 3A and 3B**). We performed a differential comparison of *APOER2* transcripts identified in the control and AD parietal cortex and identified two full-length transcripts as different between the two groups (**Fig 3C**), with both isoforms present in control but absent in AD. The first isoform, PB.97.1158, demonstrated inclusion of exon 6B and exclusion of exons 8, 15, and 18 (+ex6B, Δex8, Δex15, Δex18). This would generate an isoform at the protein level that remains in frame and includes the furin cleavage site, but lacks the second EGF-like precursor repeat, the receptor glycosylation domain, and the cytoplasmic insert. The second isoform, PB.97.1196, demonstrated inclusion of exon 6B and exclusion of exons 5, 6 and 18 (Δex5-6, +ex6B, Δex18), which remains in frame at the protein level. This isoform adds the furin cleavage site, but excludes four LDLa ligand binding repeats, and the cytoplasmic insert. To visualize how full-length *APOER2* isoforms compare between control and AD in the parietal cortex, we graphed the ranked median TPM value for each isoform in AD against control. This comparison highlights a subset of isoforms that are ranked highly in both control and AD, as well as several isoforms that are more prevalent in either group (**S2A Fig**).

We next examined the top 10 isoforms for each sample in the hippocampus. Similar to the parietal cortex, the top 10 isoforms remain relatively consistent across all six samples regardless of AD status, although with some individual variation in the precise order of abundance (**Fig 3D**). There were two isoforms that were included in the top 10 list in the parietal cortex, but not in the hippocampus, and three vice versa. Therefore, we analyzed whether those five isoforms were present in the other brain region just at lower abundances as listed in **S6 Table**. This highlighted two isoforms of interest, *APOER2* Δex4-5, +ex6B, Δex15, and *APOER2* Δex5, Δex15. *APOER2* Δex4-5, +ex6B, Δex15 is abundant in the parietal cortex, but low in the hippocampus, and trends towards being more expressed in the AD parietal cortex compared to control. *APOER2* Δex5, Δex15 was not identified in the parietal cortex, but was abundant in the hippocampus.

When we compared *APOER2* isoforms between control and AD in the hippocampus, two transcripts were different, with both being present in control and largely absent in AD (**Fig 3E**). The first isoform, PB.79.1318, demonstrated inclusion of ex6B and exclusion of ex8 and ex18 (+ex6B, Δex8, Δex18), adding the furin cleavage site, and excluding the second EGF precursor-like repeat, and cytoplasmic insert. The second isoform, PB.79.1172, also included ex6B, but excluded ex4, ex5 and ex18 (Δex4-5, +ex6B, Δex18), which adds the furin cleavage site, and removes four LDLa ligand binding repeats, and the cytoplasmic insert. Both isoforms appear to remain in frame if the canonical ATG site is used in exon 1. To visualize how *APOER2* isoforms compare between control and AD in the hippocampus, we plotted the ranked median TPM value for each isoform in AD against control. Similar to the parietal cortex, we observed a general agreement in rank between the two groups, with some outliers and isoforms more specific to one group (**S2B Fig**).

### *APOER2* exhibits differential exon inclusion and full-length transcripts in the AD parietal cortex and hippocampus

**Fig 4:**
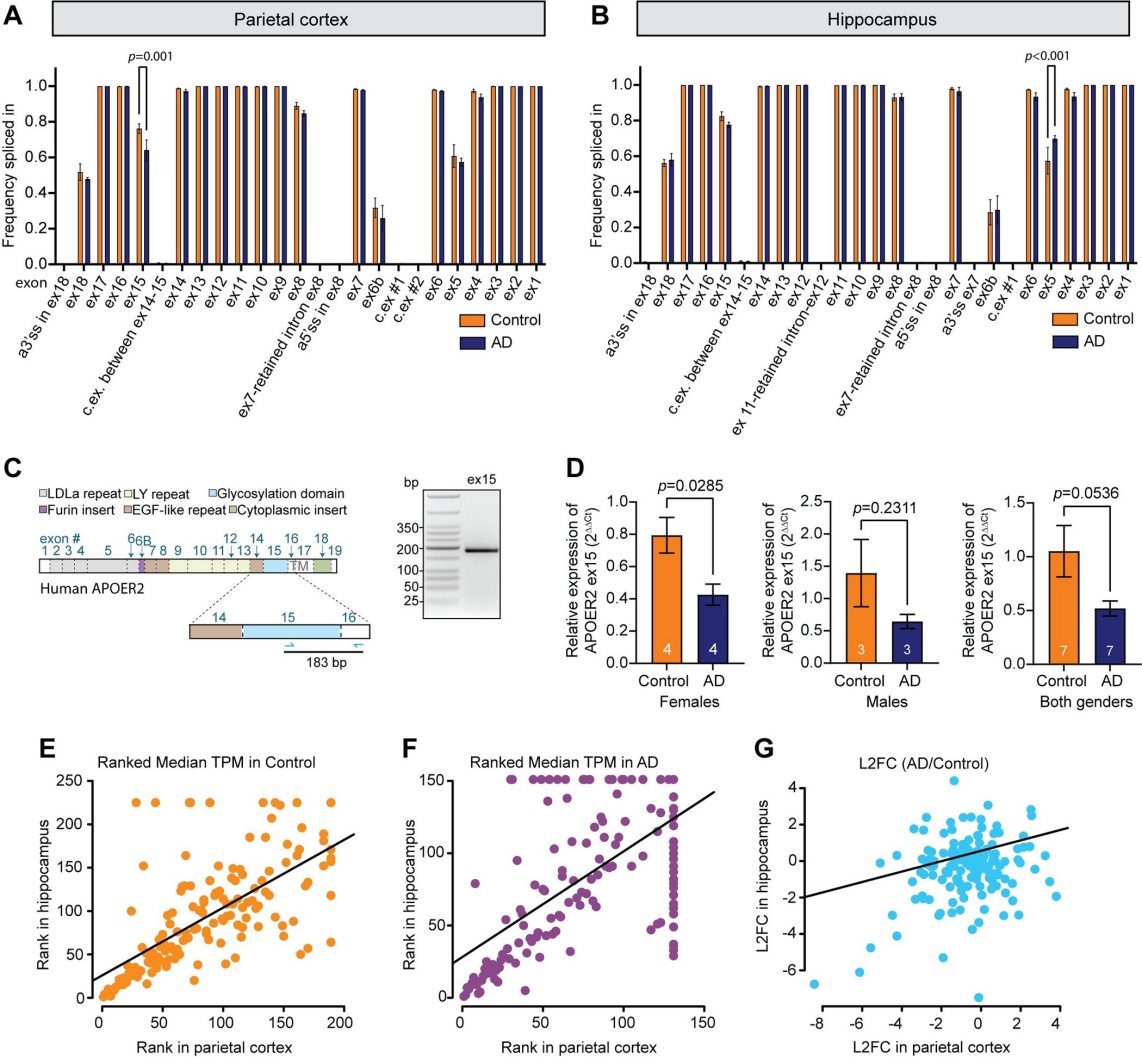
Individual splicing of cassette exons in *APOER2* is disrupted in AD. (A) *APOER2* ex15 inclusion is downregulated in the parietal cortex in AD compared to control. Bar graph depicting average frequency spliced in value for each exon in control and AD groups. (B) *APOER2* ex5 inclusion is upregulated in the hippocampus in AD compared to control. Bar graph depicting average frequency spliced in value for each exon in control and AD. For A-B, data are expressed as mean ± S.E.M and significance was determined using a 2-way ANOVA with Sidak’s multiple comparisons correction with *p*-values listed above. (C) Schematic depicting human APOER2 protein domains and corresponding coding exons along with RT-PCR strategy. On the right is a gel depicting RT-PCR of mRNA from the human parietal cortex examining inclusion of *APOER2* ex15. The PCR product was purified and sequenced, see **S4 Fig**. (D) Quantitative RT-PCR analysis of *APOER2* ex15 inclusion of *APOE* ε3/ε3 control and AD parietal cortex samples. Mean ± S.E.M. is depicted and significance was determined using a Student’s *t*-test with *p*-values listed above. (E-F) Scatterplots of the ranked median *APOER2* TPM for the (E) control or (F) AD samples in hippocampus versus parietal cortex. Only isoforms common between the two regions were graphed. (G) Scatterplot of the log_2_ fold change (L_2_FC) of AD/control *APOER2* isoforms common to the parietal cortex and hippocampus. Numbering on the plot points indicates a transcript number randomly assigned and does not indicate the rank value. Transcript numbering is comparable between E, F & G.

As each of the significantly different *APOER2* transcripts we identified demonstrate unique combinations of cassette exon skipping patterns, we calculated a frequency spliced in value for each individual exon to determine whether any individual exons demonstrate altered inclusion values across the entire isoform pool. We found that ex15, encoding the receptor glycosylation domain, demonstrates significantly less inclusion in AD compared to control in the parietal cortex, *p* = 0.001 (**Fig 4A**). As ex15 encodes the main glycosylation domain of the receptor which affects the rate of extracellular cleavage [18,19], less inclusion of ex15 could lower APOER2 levels at the cell surface where APOER2 performs its physiological functions.

Like the parietal cortex, we assessed whether inclusion of any individual exons differed between control and AD samples in the hippocampus by calculating a frequency spliced in value for each exon across all the isoforms. Exon 5 (ex5) demonstrated significantly more inclusion in AD compared to control, *p* < 0.001 (**Fig 4B**), which encodes three LDLa ligand binding repeats. We did, however, notice that one of our AD samples, AD#1, had a high abundance isoform with 6320 full-length reads associated with it that excluded ex5 but was not present in any other samples. This isoform was therefore excluded from our analysis based on the applied filter requiring isoforms be present in at least two out of three samples in at least one of the groups. The identified isoform also had an 11 base pair insertion after ex17 in *APOER2*, which aligns to the intronic sequence just before ex18, indicating a potential alternative 3’splice site in this sample (**S3 Fig**). Since this transcript was of such high abundance and was not included in the final frequency spliced in analysis, it is likely that if included, the difference in ex5 inclusion would not reach significance between control and AD. This highlights the caveats associated with long-read sequencing data, and how viewing isoforms in the context of their full-length as opposed to individual exonic makeup paint different pictures that must be combined to fully reflect accurate isoform biology. We noticed no such obvious instances of high abundance isoforms unique to an individual sample in the parietal cortex data.

To confirm the frequency of inclusion of *APOER2* ex15 in the parietal cortex, which was previously demonstrated to have reduced inclusion in AD compared to controls (**Fig 4A**), we designed RT-PCR primers targeting *APOER2* ex15 (**Fig 4C**). We performed RT-PCR on cDNA synthesized with oligo-dT primers and verified that the single 183 bp band observed in human parietal cortex samples indeed contains ex15 through sequencing (**Figs 4C** and **S4**). We expanded our analysis to include additional human parietal cortex samples from individuals homozygous for *APOE* ɛ3/ɛ3 and conducted quantitative PCR (qPCR) to assess *APOER2* ex15 inclusion. We found a significant reduction in *APOER2* ex15 inclusion in the parietal cortex of female *APOE* ɛ3/ɛ3 AD brains compared to control *p* = 0.0285, with a similar decreasing trend observed in male *APOE* ɛ3/ɛ3 AD brains, *p* = 0.2311 and those of both genders, *p* = 0.0536 (**Fig 4D**). However, we were unable to design primers validating *APOER2* ex5 inclusion in the hippocampus, as ex5 encodes several LDLa binding repeats that exhibit high sequence similarity with other exons in the *APOER2* transcript, complicating specific primer design.

### *APOER2* brain region specific and conserved isoforms

After observing differences between the parietal cortex and hippocampus, we compared the 151 isoforms that were found in both brain regions by plotting rank-transformed median TPM of each isoform in parietal cortex against hippocampus for control and AD cases separately (**Fig 4E–4G**). We found that in control, there seems to be more isoform diversity in both brain regions (**Fig 4E**), whereas in AD there is a subset of isoforms that correlate well between regions and another subset of isoforms that are present in one region yet absent in the other (**Fig 4F**).

To understand whether *APOER2* isoforms changed in the same way in AD compared to control in both brain regions, we plotted the log_2_-fold change (log_2_FC) of the 151 shared isoforms in AD compared to control in the hippocampus against the log_2_FC in the parietal cortex. Our results indicate the shared isoforms largely cluster around a log_2_FC between 1 and -1 (**Fig 4G**), suggesting *APOER2* isoforms in common between the two regions may not be strongly affected in AD compared to control.

### *APOER2* isoforms exhibit altered receptor properties that are dependent on unique alternative exon combinations

To determine whether combinatorial cassette exon splicing in *APOER2* affects receptor biology, we selected a subset of *APOER2* isoforms for functional analysis (**Table 1**). We selected three isoforms present in the top 10 isoforms in both the parietal cortex and the hippocampus with only one cassette exon alternative splicing event along with the canonical *APOER2*-FL isoform (*APOER2* Δex18, *APOER2* Δex5, and *APOER2* +ex6B). Since the hippocampus is affected earlier in AD, we selected the two isoforms we identified as having significantly more full-length reads in control compared to AD hippocampus (*APOER2* Δex4-5, +ex6B, Δex18 and *APOER2* +ex6B, Δex8, Δex18). We selected one isoform only detected in the AD hippocampus, *APOER2* +ex6B, Δex14, Δex18, which encodes the furin cleavage site and excludes the third EGF precursor-like repeat, and cytoplasmic insert, respectively. Lastly, we included the *APOER2* Δex4-5, +ex6B, Δex15 as it was detected in high amounts in the parietal cortex, and notably had low level of reads in AD in the hippocampus. Furthermore, *APOER2* Δex4-5, +ex6B, Δex15 shares a similar exon pattern to Δex4-5, +ex6B, Δex18, which was only found in control hippocampus compared to AD hippocampus, highlighting how exon inclusion patterns along the full -length of the receptor exhibit different relative read abundances in AD.

**Table 1:** *APOER2* isoforms selected for functional analysis.

Exon Annotation	Parietal Cortex	Hippocampus
FL	Most abundant	Most abundant
Δex18	2^nd^ most abundant	2^nd^ most abundant
Δex5	3^rd^ most abundant	5^th^ most abundant
+ex6B	8^th^ most abundant	4^th^ most abundant
Δex4-5, +ex6B, Δex18	Present	Significantly different (Control > AD)
+ex6B, Δex8, Δex18	Present	Significantly different (Control > AD)
Δex4-5, +ex6B, Δex15	Abundant overall, trends higher in AD	Low level of reads
+ex6B, Δex14, Δex18	Present, trends higher in AD	Unique to AD

**Fig 5:**
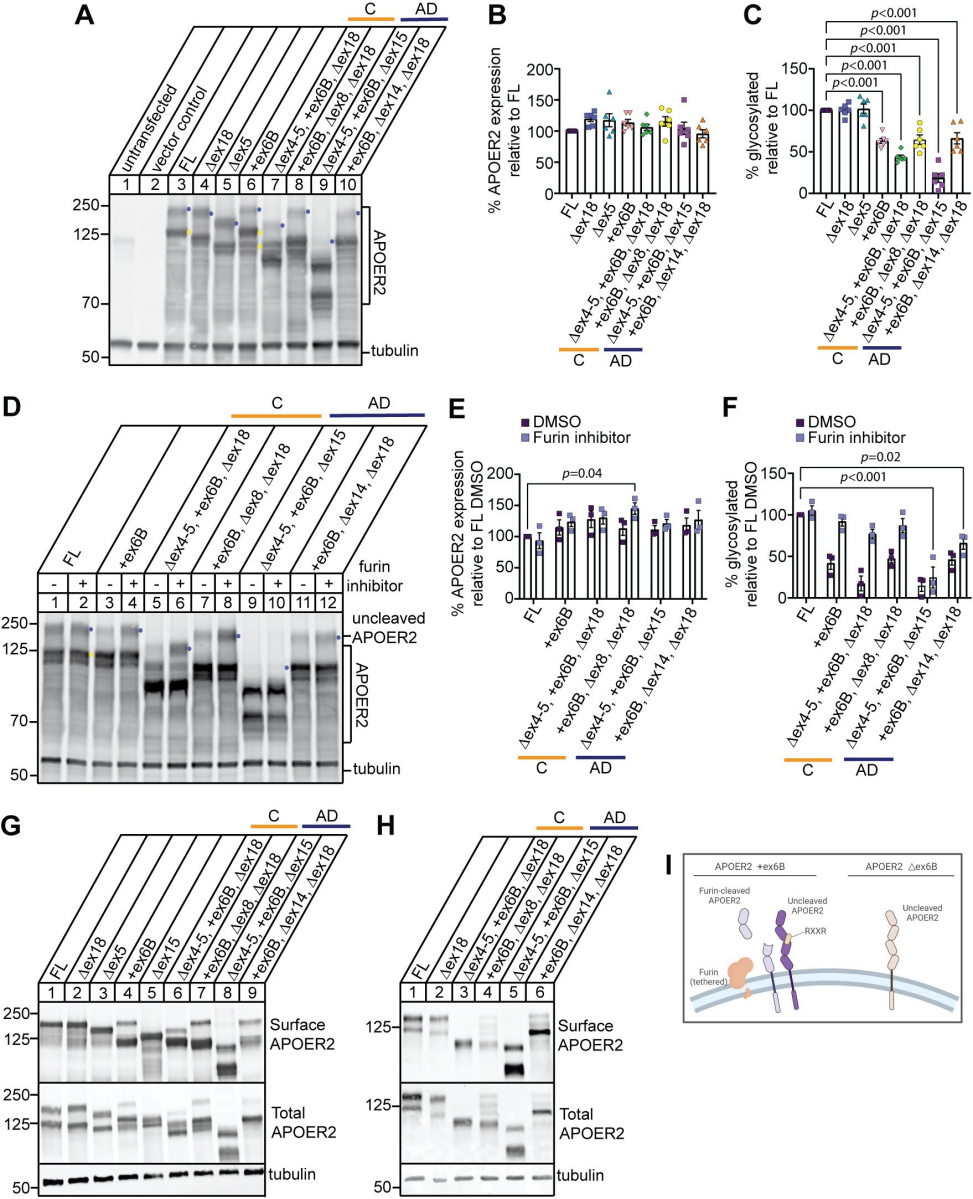
APOER2 isoforms exhibit specific changes in glycosylated receptor properties. (A) Representative immunoblots of HEK293T cells expressing APOER2 isoforms of interest and probed for APOER2 and tubulin protein expression. Purple and yellow dots indicate the mature and immature glycosylated form of APOER2, respectively. (B) Quantification of total APOER2 protein normalized to tubulin and expressed as percentage of APOER2 full-length (FL) isoform. (C) Quantification of the glycosylated APOER2 (topmost band in each lane, indicated by purple dot in A) relative to total APOER2 protein and expressed as percentage of APOER2-FL isoform. (D) Furin inhibition does not rescue AD specific APOER2 isoform glycosylation levels. Representative immunoblots of cell lysate from HEK293T cells transfected with various APOER2 isoforms and treated with either vehicle or 15 μM furin inhibitor for 24 hours. Lysate was probed for APOER2 and tubulin protein. (E) Quantification of total APOER2 expression normalized to tubulin and expressed as percentage of APOER2-FL treated with DMSO. (F) Quantification of the glycosylated topmost APOER2 band in D denoted by purple dot relative to total APOER2 protein and expressed as a percentage of APOER2-FL treated with DMSO. Data are expressed as mean ± S.E.M. (n = 3 independent experiments). Statistical significance was determined using a one-way ANOVA with Dunnett’s multiple comparisons test with *p*-values are listed above each graph. (G) APOER2 ex6B isoforms demonstrate multiple forms at the cell surface. Representative immunoblots of total protein and surface protein from HEK293T cells transfected with APOER2 isoforms of interest for 24 hours and blotted for APOER2 and tubulin (n = 4 independent experiments). (H) APOER2 ex6B isoforms predominantly express the furin cleaved form of the receptor at the cell surface. Representative immunoblots of total protein and surface protein from *Apoer2* knockout primary murine cortical neurons rescued with lentivirus expressing *APOER2* isoforms of interest and blotted for APOER2 and tubulin (n = 3 independent experiments). (I) Schematic of APOER2 receptors at the cell surface with and without ex6B (Δex6B) inclusion. Created with biorender.com.

To examine the subset of *APOER2* isoforms (listed in **Table 1**), we cloned all *APOER2* isoforms into expression plasmids individually, and transfected them into HEK293T cells to examine APOER2 protein expression. An antibody directed against the carboxyl terminus of APOER2 from HEK293T cell lysates transfected with APOER2-FL detected two APOER2 bands where the upper band is the mature glycosylated form and the lower band is the immature form (indicated by the purple and yellow dot in lane 3, respectively, **Fig 5A**). We next measured APOER2 protein level expression across the different subset of APOER2 isoforms normalized to tubulin and compared to APOER2-FL and found APOER2 isoforms were expressed at similar levels (**Fig 5B**). Since APOER2 ex6B encodes a furin cleavage site, we detected more bands in HEK293T cells transfected with APOER2 isoforms containing ex6B (indicated in lane 6, **Fig 5A**) compared to the canonical two bands normally observed in APOER2-FL. This indicates that APOER2 is likely cleaved by furin, a ubiquitously expressed protease, producing several APOER2 cleaved products. We observed APOER2 isoforms containing ex6B showed differing levels of the uppermost glycosylated receptor band and therefore quantified the percentage of glycosylated top band over total APOER2. APOER2 isoforms containing ex6B demonstrated significantly less of the uppermost receptor band compared to APOER2-FL, *p* < 0.001 (**Fig 5C**). APOER2 Δex4-5, +ex6B, Δex15 that is unique to AD showed the most striking loss in the uppermost glycosylated receptor band (indicated in lane 9, **Fig 5A** and **5C**) which is largely due to exclusion of ex15 that encodes the glycosylation domain.

We next asked whether furin inhibition could rescue the expression of the upper mature receptor band of APOER2. We transfected HEK293T cells with APOER2 isoforms containing ex6B individually using APOER2-FL as control and treated with 15 μM furin inhibitor or DMSO vehicle for 24 hours (**Fig 5D**). Furin inhibition had no effect on APOER2-FL, as expected (lane 2, **Fig 5D**). Also, furin inhibition did not alter overall total APOER2 levels across the APOER2 isoforms except for one isoform, +ex6B, Δex8, Δex18, *p* = 0.04 (**Fig 5E**). However, furin inhibition rescued the mature upper band of APOER2 +ex6B isoform similar to APOER2-FL (lane 4 in **Fig 5D and 5F**). Both APOER2 Δex4-5, +ex6B, Δex18 and APOER2 +ex6B, Δex8, Δex18 isoforms, found in human control brain samples, also demonstrated rescue of mature receptor relative to APOER-FL levels. In contrast, neither APOER2 Δex4-5, +ex6B, Δex15 or APOER2 +ex6B, Δex14, Δex18, found in the human AD brains we analyzed, were rescued back to APOER2-FL levels of mature receptor following furin inhibition, *p* < 0.001 and *p* = 0.02, respectively (lanes 10 and 12 in **Fig 5D and 5F**). Since APOER2 Δex4-5, +ex6B, Δex15 lacks the O-linked glycosylation region encoded by ex15 which is necessary for receptor trafficking to the cell membrane, the lack of rescue is likely driven by the simultaneous alternative splicing of ex15 in the isoform. This demonstrates that combinatorial splicing across APOER2 affects overall receptor biology.

Furin is a protease that is expressed in the trans-Golgi network (TGN) and tethered at the cell surface, which can cleave proteins at either location or in the endosomal trafficking pathway [20]. For example, LDLR family member LRP1 requires cleavage by furin in the TGN to fully mature [21]. Furin inhibition increased the amount of the uppermost band of APOER2, typically thought of as the mature version of the receptor that is at the cell surface. Therefore, we asked whether APOER2 isoforms containing ex6B may be cleaved at the cell surface, creating two surface APOER2 variants of each isoform. To address this, we transfected APOER2 isoforms individually into HEK293T cells and incubated with sulfo-NHS-LC-Biotin to label cell-surface proteins, and performed a surface biotinylation assay to measure cell surface APOER2. Biotinylated surface proteins were then precipitated and immunoblotted for APOER2. We detected only the upper mature glycosylated form for APOER-FL, APOER2 Δex18 and APOER2 Δex5 (lanes 1–3, **Fig 5G**). In contrast, we detected two APOER2 surface forms when ex6B was present (lane 4, **Fig 5G**). We included APOER2 Δex15 as control, since it lacks the glycosylation domain of the receptor and is also among the top 10 isoforms present in both the parietal cortex and the hippocampus (lane 5, **Fig 5G**). This suggests APOER2 may be cleaved at the cell surface by furin creating two surface APOER2 variants, cleaved and uncleaved, that can bind ligands and perform other receptor functions (schematized in **Fig 5I**). It is also possible that APOER2 is cleaved by furin in the TGN or the endosomal pathway, yet still trafficked to the cell surface as a cleaved product. To address whether similar APOER2 cleavage events occur in neurons, we infected primary *Apoer2* knockout neurons with lentiviral human APOER2 variants and found similar banding patterns with the human AD APOER2 variants when ex6B is present where the furin-cleaved receptors were dominant at the surface, except for APOER2 +ex6B, Δex14, Δex18 isoform (**Fig 5H**) which may differ likely due to cell-type specificity in glycosylation and protein trafficking.

### *APOER2* isoforms exhibit differential APOE-mediated receptor processing

**Fig 6:**
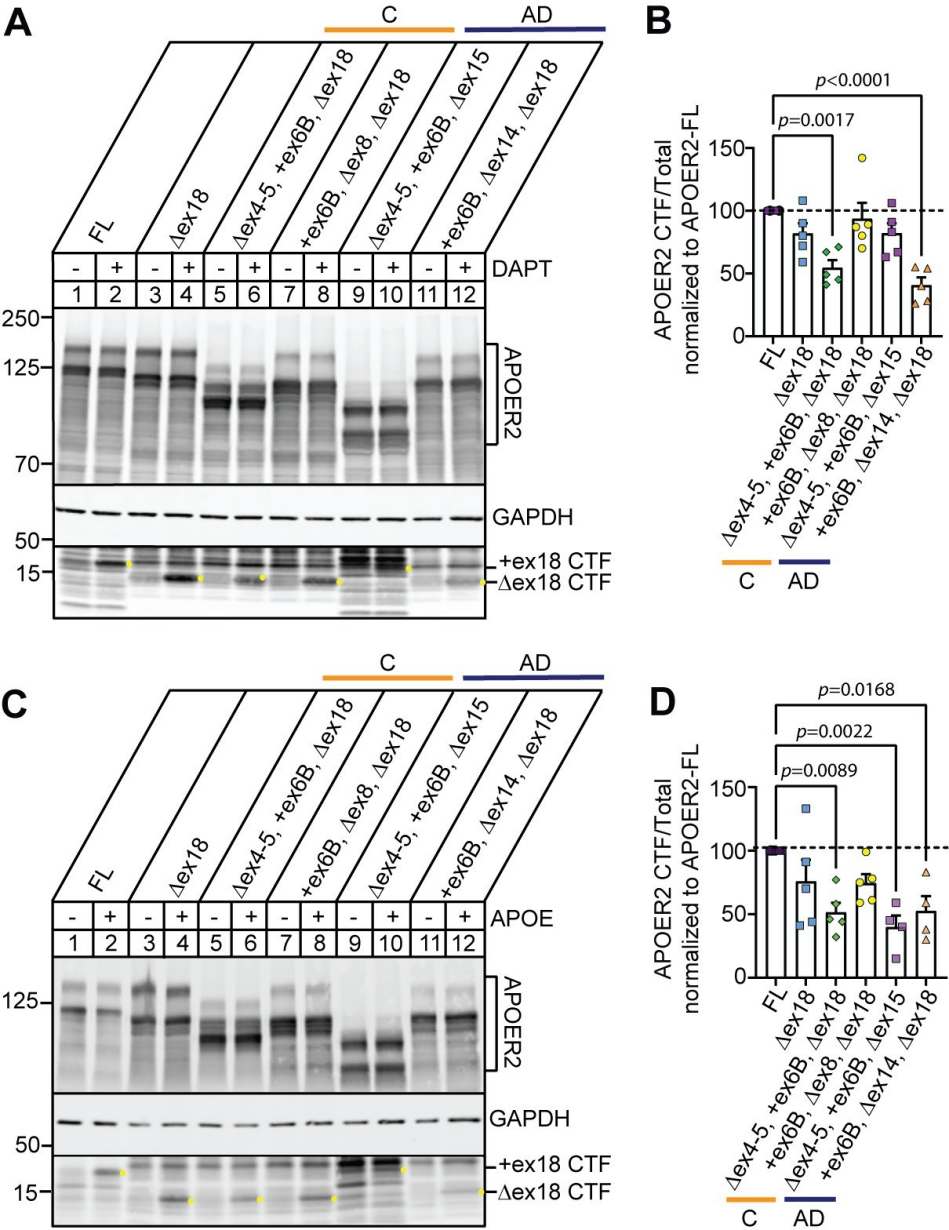
APOER2 isoforms exhibit differential APOE-mediated receptor cleavage. (A) Representative immunoblots of APOER2 isoforms of interest expressed in HEK293T cells and treated with γ-secretase inhibitor DAPT for 24 hours to measure APOER2 CTF accumulation, indicated by yellow dot. (B) Quantification of APOER2-CTF normalized to each APOER2 variant (total) and expressed as a percentage of APOER2-FL treated with DAPT (n = 5 independent experiments). (C) Representative immunoblots of APOER2 isoforms of interest expressed in HEK293T cells and treated with APOE mimetic peptide for 30 minutes to measure APOER2 CTF accumulation, indicated by yellow dot. (D) Quantification of APOER2-CTF normalized to each APOER2 variant (total) and expressed as a percentage of APOER2-FL treated with APOE (n = 4–5 independent experiments). Data are expressed as mean ± S.E.M. Statistical significance was determined using a one-way ANOVA with Dunnett’s multiple comparisons test and *p*-values are listed above each graph.

APOER2 is also sequentially cleaved at the cell membrane by enzymes other than furin. APOER2 is cleaved in the extracellular region first by α-secretases, leaving behind a membrane-bound carboxyl terminal fragment (CTF) that is subsequently cleaved by γ-secretase to release an intracellular domain (ICD) [18,19,22], which translocates to the nucleus to activate an enhancer program critical for transcription of learning and memory genes [23]. To determine whether APOER2 variants affect receptor cleavage patterns, we transfected APOER2 isoforms individually in HEK293T cells and treated with 2 μM DAPT, a γ-secretase inhibitor for 24 hours. Inhibition of γ-secretase allows accumulation of APOER2-CTF as a proxy for ICD generation, as the ICD is too small and difficult to resolve by immunoblotting. As expected, inhibition of γ-secretase leads to accumulation of the APOER2-CTF (**Fig 6A**). When we compared APOER2-FL with APOER2 Δex18, lacking 59 residues in the cytoplasmic domain, we detected a decrease in the size of CTF, consistent with the exclusion of ex18 (lanes 3–4, **Fig 6A**). We next sought to determine whether the combinational diversity in the APOER2 ligand-binding domains affect APOER2 processing. We found APOER2 Δex4-5, +ex6B, Δex18 isoform (unique to control) and APOER2 +ex6B, Δex14, Δex18 isoform (unique to AD) generated lower amounts of CTFs compared to APOER2-FL (45.4% and 59.4% decrease, *p* = 0.0017 and *p* < 0.0001 respectively) (**Fig 6A and 6B**), suggesting that APOER2 splice variants display differential cleavage events.

Next, we tested whether APOER2 cleavage by γ-secretase can be induced in a ligand-regulated manner by binding APOE. We have previously shown that APOE mimetic peptide (133–149 residues) derived from the receptor binding region, influences APOER2 splice variant receptor processing [15]. HEK293T cells were transfected with individual APOER2 isoforms for 24 hours and treated with 50 μM of APOE mimetic peptide. Cell lysates were collected and processed for detection of APOER2-CTFs. Addition of APOE induced an accumulation of CTF generation in APOER2-FL and similarly in APOER2 Δex18 (lanes 2 and 4, **Fig 6C**). We found APOER2 Δex4-5, +ex6B, Δex18 isoform (unique to control) and APOER2 +ex6B, Δex14, Δex18 isoform (unique to AD) generated lower amounts of CTFs compared to APOER2-FL following APOE treatment (48.6% and 47.5% decrease, *p* = 0.0089 and *p* = 0.0168 respectively, **Fig 6D**). Interestingly, there was a 60% decrease of CTF generation with APOER2 Δex4-5, +ex6B, Δex15 (unique to AD) compared to APOER2-FL in response to APOE, *p* = 0.0022 (**Fig 6D**). This is most likely explained by the simultaneous splicing of ex15 which leads to a reduction in mature glycosylated receptor at the surface to interact with APOE. Since APOER2 and the generation of APOER2-ICD (where CTF serves as a substrate) are necessary for critical functions including transcription of learning and memory genes, our data suggests that APOE-mediated interaction with APOER2 isoforms elicits differential receptor processing that may alter downstream APOE-mediated signaling events.

### *APOER2* isoforms display altered synaptic changes in primary murine neurons

**Fig 7:**
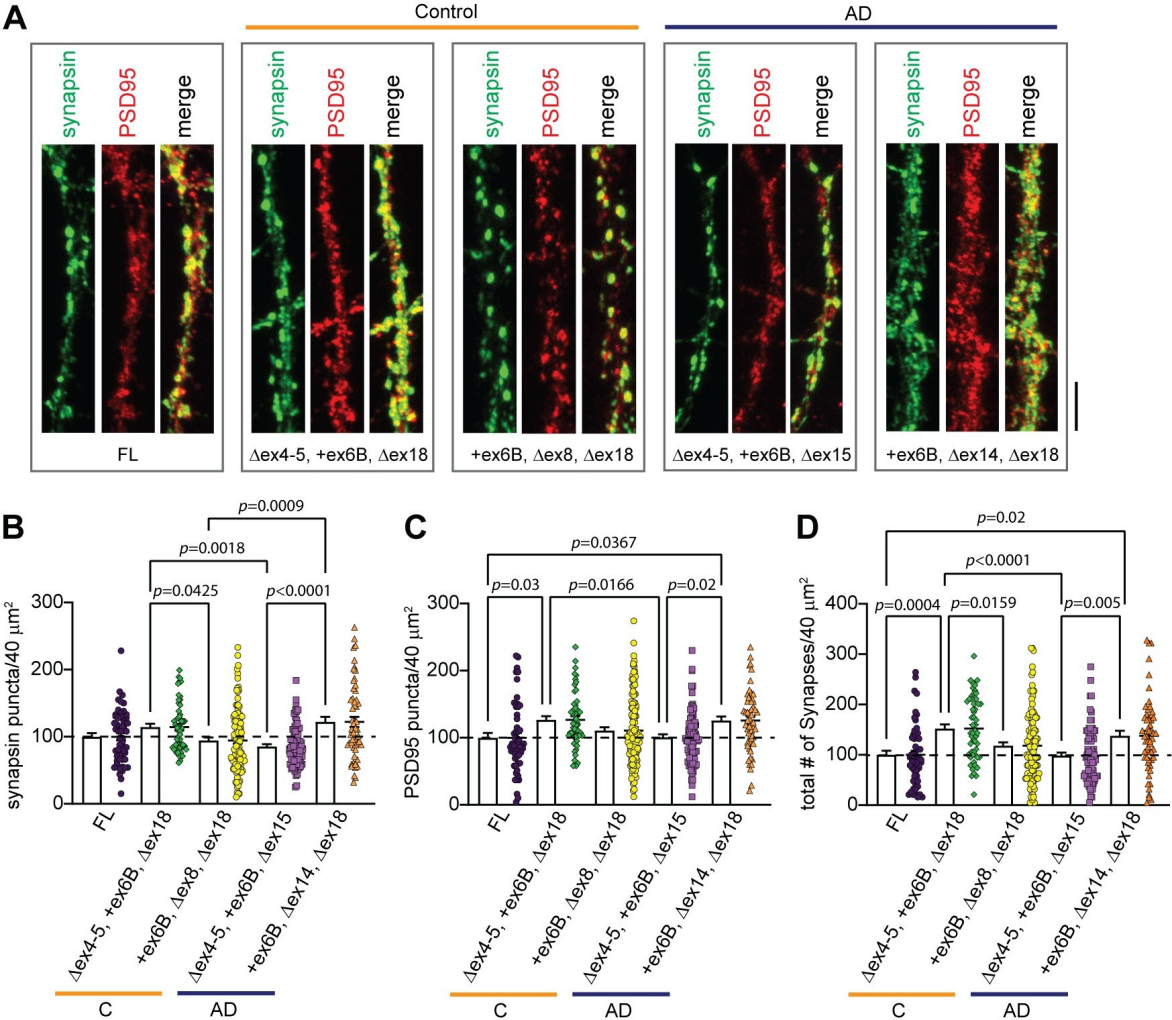
APOER2 isoform unique to AD leads to a change in total synapse number. (A) Representative images of hippocampal neuronal processes of *Apoer2* homozygous mouse knockout neurons infected with human APOER2-FL, APOER2 Δex4-5 +ex6B Δex18, APOER2 +ex6B, Δex8, Δex18, APOER2 Δex4-5 +ex6B Δex15 or APOER2 +ex6B, Δex14, Δex18 lentivirus and stained with synapsin (green) and PSD95 (red) at 14 DIV. Scale bar, 5 μm. (B) Bar graph of quantification in the number of synapsin puncta. (C) Quantification of the number of PSD95 puncta. (D) Quantification of synapsin and PSD95 colocalization in neuronal processes depicting the number of total synapses. Each data point represents the average number of surface puncta greater than 10 voxels per region of interest for each captured neuron. N = 2 independent experiments. Data are expressed as mean ± S.E.M. Statistical significance was determined using a one-way ANOVA with Tukey’s multiple comparisons test and *p*-values are listed above each graph.

Since APOER2 isoforms have been shown to modify synapse number and function [14,15], we performed immunofluorescence labeling using antibodies against the presynaptic protein synapsin and the postsynaptic marker PSD95 on *Apoer2* knockout mouse neurons rescued with lentiviral human APOER2-FL, APOER2 variants found in human control (APOER2 Δex4-5, +ex6B, Δex18, APOER2 +ex6B, Δex8, Δex18) or AD brains (APOER2 Δex4-5, +ex6B, Δex15, APOER2 +ex6B, Δex14, Δex18 isoforms). We consistently titer the lentivirus to achieve receptor expression levels that mirror endogenous levels (**S5 Fig**) and measured the number of synapsin and PSD95 puncta independently and the number of synapses defined by the colocalization of synapsin and PSD95 (**Fig 7**). When we measure the number of PSD95 puncta in neurons, the control APOER2 Δex4-5, +ex6B, Δex18 variant had a 26.6% increase compared to neurons infected with APOER2-FL (*p* = 0.03) which reflected a 52% increase in the total number of synapses (*p* = 0.0004) that was not observed with the other control APOER2 +ex6B, Δex8, Δex18 variant (**Fig 7A, 7C and 7D**).

Neurons infected with the APOER2 Δex4-5, +ex6B, Δex15 variant that was found unique to AD brain exhibited a decreased in the number of synapsin (*p* = 0.0018) and PSD95 (*p* = 0.0166) puncta compared with control-specific APOER2 Δex4-5, +ex6B, Δex18 culminating to a 54% decrease in the total number of synapses (*p* < 0.0001, **Fig 7A** and **7D**). In contrast, we found a 38% increase in total number of synaptic puncta when we compared neurons infected with the other AD APOER2 +ex6B, Δex14, Δex18 variant compared to APOER2-FL (*p* = 0.02). Altogether, these results suggest that APOER2 isoforms display altered synaptic changes in primary murine neurons.

## Discussion

Our study reveals that a multitude of full-length *APOER2* isoforms exist in the human brain, specifically in the hippocampus and parietal cortex, as determined by single molecule, long-read RNA sequencing. In our analysis, we identified over 200 unique *APOER2* isoforms in these regions from individuals with AD and age-matched controls (**Figs 1 and 2**). In the parietal cortex, 183 isoforms were common to both the control and AD groups, with 20 isoforms exclusive to the control group and 6 unique to the AD group. Similarly, in the hippocampus, we found 207 isoforms shared between both groups, alongside 37 isoforms specific to the control group and 5 unique to the AD group. These results indicate that the pattern of *APOER2* alternative splicing exhibits variations in AD compared to controls in both brain regions studied.

We identified four full-length *APOER2* transcripts with significant differences between the control and AD groups, two each in the parietal cortex and hippocampus (**Fig 3**). These four isoforms each contain alternative splicing of exons that encode functional domains in the final protein and are predicted to have functional impacts. We also found disruptions in the alternative splicing of individual cassette exons in *APOER2* in AD. Notably, in the parietal cortex, there was a higher frequency of exclusion of ex15, which encodes APOER2’s glycosylation domain, in AD compared to control group (**Fig 4**). We performed qPCR and confirmed a significant reduction in *APOER2* ex15 inclusion in the parietal cortex of female *APOE* ε3/ε3 AD brains. Conversely, in the hippocampus, ex5 which encodes three LDLa ligand binding repeats, showed increased inclusion in AD relative to controls. These findings are interesting, as both of these exons can be skipped in-frame and encode distinct functional domains. However, given the limited sample, coupled with the previously mentioned isoform specific to sample AD#1 in the hippocampus (**S3 Fig**), requires validation of these results in a larger cohort. Additionally, pairing long-read sequencing with concurrent RNAseq data could further validate splice junctions and quantify the expression levels of different exons. Interestingly, we did not detect changes in ex18 inclusion between AD and control groups which have been previously reported [13]. This difference could be attributed to region-specific variations, as previous studies observed changes in ex18 inclusion in the middle temporal cortex of AD brain samples [13].

We also examined how combinatorial cassette exon splicing in *APOER2* might impact receptor biology. We found that *APOER2* isoforms containing ex6B, which encodes a furin cleavage site, exhibited a decrease in the glycosylated upper band that can be rescued by furin inhibition in certain *APOER2* isoforms, depending on the combination of alternative splicing events across the transcript (**Fig 5**). Interestingly, the isoforms that were not rescued by furin inhibition were predominantly found in the AD group (specifically, APOER2 isoforms Δex4-5, +ex6B, Δex15 and APOER2 +ex6B, Δex14, Δex18). Our results suggest that furin-cleaved APOER2 is present on the cell surface and may retain the capacity to bind ligands, given its preservation of EGF-precursor like repeats and the β-propeller domain. In the context of AD, the activity of furin could be modified by increased calcium levels [24], potentially affecting its ability to cleave APOER2 ex6B-containing receptors in the AD brain. Furin is also known to be involved in the proteolytic processing of enzymes related to Aβ generation, such as ADAM10 and BACE1, which are responsible for α- and β-APP cleavage events, respectively [20]. Furthermore, APOER2 has been implicated in Aβ production *in vitro* through its interaction with APOE, and intracellular adaptor proteins APBA1 and APBA2, which bind to ex19 (ex18 in humans) and APP itself [25]. This presents a complex model of receptor processing in AD, where altered *APOER2* splicing and the modified activity of enzymes involved in both APOER2 and APP processing are interconnected and functionally affected. These factors may, in part, contribute to the pathogenic receptor processing and signaling observed in AD.

APOER2 undergoes cleavage by secretase enzymes, resulting in the generation of CTF and ICD generation that can translocate to the nucleus and modify epigenetic signature of transcripts related to learning and memory, a process that is dependent on Reelin binding [23]. Our study demonstrates that the diversity in combinatorial splicing of APOER2’s ligand-binding domains influences its processing, indicating that different APOER2 splice variants may exhibit varied receptor cleavage patterns. This variation could also affect the role of APOE in modulating transient APOER2 cleavage (**Fig 6**). We observed a notable decrease in CTF generation in response to APOE for both APOER2 variants identified in the AD group. Specifically, the reduced CTF generation in the APOER2 variant Δex4-5, +ex6B, Δex15 can be attributed to the concurrent exclusion of ex15, which likely leads to a decreased presence of mature glycosylated receptors at the surface available for interaction with APOE. Given that glycosylation of APOER2 is thought to regulate ICD generation [19], the altered cleavage patterns we noted at the extracellular level could impact APOE-APOER2 biology. The interaction between ligands and receptors like APOER2 is not solely based on binding to individual ligand-binding repeats. Exon skipping not only removes specific domains but also alters the overall structure and folding of the receptors. Our findings indicate that certain exon skipping events in APOER2 enhance CTF formation, while others reduce it. This suggests that variations in exon inclusion are crucial in determining the functional outcomes of these interactions.

Because altered memory is a critical phenotypic component of AD, understanding the impact of alternative splicing of *APOER2* on neuronal and synaptic processes is crucial. Here, we observed notable changes in presynaptic synapsin puncta, postsynaptic PSD95 puncta and total synapse number in primary dendrites expressing APOER2 variants that were exclusive to either the AD or age-matched control groups (**Fig 7**). Future studies exploring how these AD and control-specific *APOER2* isoforms influence neuronal activities such as neuronal firing and synaptic transmission will be important to address the mechanistic links between *APOER2* splicing variations and neuronal function. Our study also revealed that the majority of *APOER2* isoforms with a high number of full-length reads exhibited consistent proportions across samples, regardless of disease status (**Fig 3**) and, to some extent, across different brain regions (**Fig 4**). This consistency suggests the presence of abundant or common *APOER2* isoforms that remain relatively stable in the brain irrespective of AD or brain region. In contrast, it appears that the isoforms present at lower levels are those that undergo changes in disease and vary between regions. Our analysis identified specific isoforms unique to the AD group compared to the control group in both the parietal cortex and hippocampus. Moreover, we observed more variation in *APOER2* isoforms between different brain regions in AD than in controls, indicating that combinatorial splicing might be dysregulated in AD, leading to the emergence of distinct, less abundant *APOER2* isoforms. This finding is interesting since splicing is known to be regulated in a spatiotemporal specific manner [26]. Therefore, these AD-specific *APOER2* isoforms might reflect changes specific to disease, regional differences, or a combination of both. Future research is needed to confirm whether these unique isoforms are translated at the protein level. Although detecting lowly expressed isoforms at the protein level can be technically challenging [27,28], emerging methods that integrate RNAseq and mass spectrometry data [29,30] could enhance the feasibility of such studies.

A notable limitation of our study is the inherent nature of AD, which is marked by a progressive loss of neurons [31]. Consequently, the changes we observed in *APOER2* isoforms might be attributed to variations in the proportions of neuronal subtypes that express specific *APOER2* isoforms. Additionally, the expression of *APOER2* is not limited to neurons since *APOER2* is also expressed by radial glia and intermediate progenitor cells [32]. Given that our study utilized bulk tissue analysis, it becomes crucial to determine whether different cell types or even individual cells within subtypes exhibit distinct patterns of *APOER2* isoform expression compared to one another, or if a single cell can express the entire range of isoforms at any given time. This will discern whether the observed differences in *APOER2* isoforms are a result of disease-specific changes within cells or due to the loss of certain cell populations. Approaches that apply long-read RNA sequencing at a single cell level [33,34] would be useful for answering these questions.

Looking ahead, it is crucial to explore how *APOER2* isoforms vary among individuals, especially between genders and across different *APOE* genotypes. Our current study focused on females with the *APOE* ɛ3/ɛ3 genotype, which reflects the demographic population since about two-thirds of AD individuals are women [35], and the ɛ3 allele is the most prevalent allele in the general population [36]. However, given that alterations in *Apoer2* splicing in mice have exhibited sex-dependent phenotypic effects [13], it is important to extend the analysis of human *APOER2* splicing to both males and females. Recent research has highlighted the intriguing aspect of splicing changes in AD, showing that alterations in proteins associated with RNA processing are linked with AD neuropathology and manifest early in the disease progression, occurring independently of both age and *APOE* genotype. This suggests that these changes might represent a distinct risk factor for AD [37,38]. Furthermore, future research should aim to uncover detailed mechanisms of ligand-receptor interactions and the biological consequences of receptor splicing and signaling. This includes exploring how Dab1, which binds to the intracellular domain of APOER2, activates the PI3K-Akt pathway which is critical for regulating tau phosphorylation and neuronal functions. Understanding these processes are essential for elucidating how these factors affect disease susceptibility and resilience in AD.

## Materials and methods

### Ethics statement

Animal studies were approved by the Institutional Animal Care and Use Committee (IACUC), protocol number, PROTO201800553 and recombinant DNA studies were approved by the Institutional Biosafety Committee, protocol number, 24–1555.

Human tissue obtained from NIH Neurobiobank was institutional approved by Boston University. Written informed consent was provided to the NIH Neurobiobank.

### RNA isolation from human post-mortem tissue

De-identified human post-mortem brain tissue was acquired through the National Institutes of Health (NIH) NeuroBioBank and stored at -80°C. Total RNA was isolated from parietal cortex and hippocampal tissue using TRIzol reagent (Invitrogen) according to manufacturer protocol. GlycoBlue Coprecipitant (Thermo Fisher Scientific) was used during isolation for visualization of RNA pellet. Final resuspension of RNA was performed with 20 μL diethyl pyrocarbonate (DEPC; Sigma) treated H_2_O. Purified RNA was quantified using a Nanodrop Spectrophotometer and diluted to a concentration between 0.05–5 ng/μL for quality assessment. RNA quality was evaluated using the Agilent RNA 6000 Pico kit for the Agilent 2100 Bioanalyzer according to manufacturer protocol. All samples demonstrated RNA Integrity Numbers (RIN) greater or equal to 5.9.

### APOER2 cDNA synthesis and RT-PCR

To generate *APOER2* cDNA amplicons, 1 μg of total RNA was incubated with APOER2 reverse primer CMG1836 (TCAGGGTAGTCCATCATCTTCAAGGC) and dNTPs at 65°C for 5 minutes, then cooled on ice for at least one minute. First strand cDNA synthesis was carried out using Superscript III Reverse Transcriptase mix (Thermo Fisher Scientific) supplemented with DTT and SUPERase RNase Inhibitor (Thermo Fisher Scientific). Mix was incubated at 55°C for 60 minutes followed by 15 minutes at 70°C. RNA was degraded by addition of 1 unit of RNase H (Thermo Fisher Scientific) and incubated at 37°C for 20 minutes.

RT-PCR was carried out off first strand cDNA in multiple 50 μL reactions using forward primer CMG1837 (ATGGGCCTCCCCGAGCC) and reverse primer CMG1836 (TCAGGGTAGTCCATCATCTTCAAGGC) with Q5 High-Fidelity DNA Polymerase (NEB) supplemented with 1 M Betaine, 3% DMSO and 5 μg BSA. Cycle utilized was: 98°C 2 minutes; 30 cycles of: 98°C- 10 seconds, 64°C- 30 seconds, 65°C- 1 minute 40 seconds; 72°C- 10 minutes. RT-PCR reactions were pooled and subject to 0.5X SPRI size selection and purification using AMPure XP beads (Beckman Coulter). cDNA amplicons were quantified using both a NanoDrop spectrophotometer and Qubit Fluorometer and submitted to either the Cold Spring Harbor Laboratory Sequencing Technologies and Analysis Shared Resource (hippocampal amplicons) or the Icahn School of Medicine at Mount Sinai Genomics Core Facility (parietal cortex amplicons) for Pacific Biosciences Isoform-Sequencing (IsoSeq) library preparation and long-read sequencing.

### PacBio targeted IsoSeq

For library preparation, equal amounts of cDNA amplicons were prepared using the PacBio express template preparation kit, and each sample went through single-strand overhang removal, DNA damage repair and end-repair by standard methods. Samples were barcoded with SMRTbell adaptors and pooled in an equimolar fashion to make two pools, each containing 3 samples. Pooled libraries were purified using 0.45X AMPure beads. Polymerase annealing and binding was performed according to standard methodology, and each library was loaded onto a Sequel II SMRTcell using standard parameters for a 3 kb library. CCS generation and barcode demultiplexing was performed using SMRTlink software. SMRTLink software was also used for initial analysis according to standard IsoSeq parameters: primer removal, identification and counting of read clusters and generation of high-quality polished isoforms.

### Analysis of PacBio single molecule, long-read RNA sequencing data

High-quality isoforms from IsoSeq pipeline for each sample were first analyzed to determine whether sample pools generated equivalent amounts of associated data. The total number of full-length reads associated with high-quality isoforms were graphed for each pool and evaluated with an unpaired *t*-test. For the parietal cortex, there was a clear pool effect between the two SMRTcells utilized; therefore, samples were randomly subsampled off the IsoSeq generated cluster report down to the number of full-length reads associated with the sample with the lowest reads (AD#3). High-quality isoforms were collapsed into unique isoforms using cDNA Cupcake v17.0.0 (https://github.com/Magdoll/cDNA_Cupcake) with a maximum 5’ and 3’ difference of 10 base pairs and without merging shorter isoforms. cDNA Cupcake was also used to generate isoform abundance reports and to filter 5’ fragments and isoforms with less than two full-length reads. cDNA Cupcake generated general feature format (gff) files for each individual sample were reference corrected against the human (hg38) genome using SQANTI3 v.2.0.0 [39]. Corrected isoforms were run through the cDNA Cupcake collapse step again to merge any duplicate isoforms post reference correction. A custom python code was written to regenerate abundance files based on the grouping file output from the second cDNA Cupcake collapse step. Individual files in an experiment were chained to identify isoforms common between samples using cDNA Cupcake’s chain isoforms script with standard parameters. Generated chained gff file was run through SQANTI3 for reference correction and transcript annotation.

Final transcript classification output from SQANTI3 was parsed in R (R Core Team, 2020) to remove any duplicate chained transcripts and to include only *APOER2* transcripts that were present in at least two out of three samples in one of the groups (Control or AD) to increase isoform confidence. For exon annotation, *APOER2* exons were extracted from the SQANTI3 gff3 output file and unique exons were extracted and manually annotated using the Broad Institute’s Integrative Genomics Viewer (IGV) [40]. A custom R script was then written to annotate the exons of individual isoforms by comparison to the extracted and annotated unique *APOER2* exons and generate a binary splice matrix [41]. Transcripts were filtered to contain only isoforms that had exons present in at least ten unique transcripts. The final list of *APOER2* transcripts were compared for differential expression using DeSeq2 [42] with a False Discovery Rate (FDR) of 0.1. Transcript maps were generated for visualization of isoforms using an adapted version of publicly available code in github [43]. To compare reads among samples in scatterplots and barplots, the full-length read counts were normalized by multiplying by one million and dividing by the total number of final *APOER2* full-length reads for that sample (transcripts per million, TPM).

### Quantitative PCR analysis

Total RNA was extracted using TRIzol reagent (Invitrogen) according to manufacturer’s protocol and purified using the RNeasy Mini Kit (Qiagen). The cDNA was synthesized using Accuris qMax cDNA Synthesis Kit (Thomas Scientific). qPCR was performed in an ABI Prism 7900HT Fast Real-Time PCR System (Applied Biosystems) using the Power SYBR Green PCR Master mix (Thermo Scientific) with a two-step cycling protocol and an annealing/extension temperature of 60°C. The experiment was performed with three technical replicates each. The relative amount of *APOER2* ex15 inclusion was normalized using *RPL13* as a housekeeping gene and the fold change in gene expression was calculated using the ΔΔCt method with the non-AD samples serving as control. Primers for human *APOER2* ex15 inclusion were 5’-CACCTACCAGAACCACAGCA-3’ (forward) and 5’-CCGATAACAGCGGCAGTGAC-3’ (reverse); human *RPL13*: 5’-CCTGGAGGAGAAGAGGAAAGAGA-3’ (forward) and 5’-TTGAGGACCTCTGTGTATTTGTCAA-3’ (reverse). Data analysis was performed using the ABI Prism 7900HT SDS Software.

### Molecular cloning

To generate pcDNA3.1-huAPOER2 Δex4-5, +ex6B, Δex18, pcDNA3-huAPOER2 5’AgeI Δex4-5, +ex6B- V5-His TOPO TA and pcDNA4/HisMax-huAPOER2 AgeI-Stop Δex18 TOPO TA were each digested with AgeI and EcoRI-HF (NEB) and the fragment containing either the 5’ or 3’ end of APOER2 was gel purified. Vector backbone pcDNA3.1/myc-His A was digested with EcoRI and the two APOER2 fragments ligated together into the backbone using T4 DNA ligase (NEB). Similarly, to generate pcDNA3.1-huAPOER2 Δex4-5, +ex6B, Δex15, pcDNA3-huAPOER2 5’AgeI Δex4-5, +ex6B- V5-His TOPO TA and pcDNA4/HisMax-huAPOER2 AgeI-Stop Δex15 TOPO TA were digested with AgeI and EcoRI-HF (NEB) restriction enzymes. Digests were run on an agarose gel, and APOER2 fragments were excised and gel purified. The 5’ and 3’ APOER2 fragments were ligated into EcoRI digested pcDNA3.1/myc-His A vector. Positive clones were screened by restriction digest and sequencing analysis to confirm orientation of insert. All sequencing analysis was performed using SnapGene6.0. HuAPOER2 +ex6B, Δex14, Δex18 was assembled using NEBuilder HiFi DNA Assembly Master Mix (NEB) to piece together three APOER2 PCR fragments into pcDNA3.1/myc-His A digested with EcoRI-HF (NEB). APOER2 exons 1–7 (including ex6B) were PCR amplified from pcDNA3.1-huAPOER2 +ex6B using primers CMG2107 (CCACTAGTCCAGTGTGGTGGGAATTCCCCGCCATGGGC) and CMG2108 (TCATCAATGTCGCCACAGGTCTTCTGGTC). APOER2 exons 8–13 and 15–19 (excluding ex18) were amplified from pcDNA3.1-huAPOER2 Δ18 using primer pairs CMG2109 (ACCTGTGGCGACATTGATGAGTGCAAGGAC) and CMG2110 (GATTGAGGTGCTCTTGGCTGCTTCAGCTC) and CMG2111 (CAGCCAAGAGCACCTCAATCTACCTCAAC) and CMG2112 (ACTGTGCTGGATATCTGCAGTCAGGGTAGTCCATCATC), respectively. Fragments were assembled according to NEBuilder HiFi DNA Assembly Master Mix (NEB) standard protocol. Colonies were screened by Sanger sequencing.

HuAPOER2 +ex6B, Δex8, Δex18 was assembled by using NEBuilder HiFi DNA Assembly Master Mix (NEB) to piece together two APOER2 PCR fragments into pcDNA3.1/myc-His A digested with EcoRI-HF (NEB). APOER2 exons 1–7 (+ex6) were amplified from pcDNA3.1-huAPOER2 +ex6B using primers CMG2107 (CCACTAGTCCAGTGTGGTGGGAATTCCCCGCCATGGGC) and CMG2113 (CTCTTGCCAGCGCCACAGGTCTTCTGGTC). APOER2 exons 9–19 (excluding ex18) were amplified from pcDNA3.1-huAPOER2 Δ18 using primers CMG2114 (ACCTGTGGCGCTGGCAAGAGCCCATCCC) and CMG2112 (ACTGTGCTGGATATCTGCAGTCAGGGTAGTCCATCATC). Fragments were assembled according to NEBuilder HiFi DNA Assembly Master Mix (NEB) manufacturer protocol. Colonies were screened by Sanger sequencing.

pFSW-IRES-GFP was assembled using NEBuilder HiFi DNA Assembly Master Mix (NEB). Briefly, IRES-GFP cassette was PCR amplified from ZPKKD30+CaM1,2,3,4-Silent plasmid using primers CMG2168 (GTCTAGAGAATTCTTCGAAACCGGTAGATCCAATTCCGCCCCC) and CMG2169 (TTGATATCGAATTGTTAACGTTACTTGTACAGCTCGTCCATG). Destination vector pFSW was digested with both AgeI and BamHI-HF (NEB) and treated with Quick-CIP (NEB) prior to HiFi DNA Assembly reaction carried out at 50°C for 15 minutes. Colonies were screened for positive clones through restriction digest and sequencing analysis. pFSW-IRES-GFP was then digested with EcoRI-HF (NEB) and dephosphorylated with Quick-CIP. APOER2 isoforms were PCR amplified from pcDNA3.1 expression plasmid using CMG2170 (ATATCGAATTCCTCGAGTCAGGGTAGTCCATC) and CMG1812 (GATATGAATTCGGCCACCATGGGCCTCCC), digested with EcoRI-HF and ligated into pFSW-IRES-GFP using T4 Ligase (NEB). Positive clones were screened by colony PCR and sequencing to determine insert orientation.

### Cell culture

HEK293T cells (ATCC CRL-3216) were cultured in Dulbecco’s Modified Eagle’s Medium (DMEM; Gibco) with 10% Fetal Bovine Serum (FBS; Atlas Biologicals) and 1% Penicillin/Streptomycin (Gibco). Cells were maintained in an incubator at 37°C with 5% CO_2_. HEK293T cells were plated in cell culture treated plates and transfected at 50–70% confluency. For biotinylation assay, HEK293T cells were plated on Matrigel (Corning) coated cell culture treated plates. FuGENE 6 (Roche) was utilized for the transfection following manufacturer protocol and using a ratio of 1:3 (DNA plasmid:FuGENE 6). Cell lysates were collected in 1X sample reducing buffer 0.05 M Tris- HCl, pH 6.8, 10% glycerol, 10% β-mercaptoethanol, 2% SDS, and 0.005% bromophenol blue 24 to 48 hours post-transfection. For the furin inhibition assay, six hours after transfection, cells were treated with 15 μM Calbiochem Furin Inhibitor I (Decanoyl-RVKR-CMK; Millipore Sigma, 344930) or equivalent DMSO (Sigma) vehicle control. To quantify APOER2 C-terminal fragment (CTF), HEK293T cells were treated with either varying concentrations of N-[N-(3,5-Difluorophenacetyl-L-alanyl)]-(S)-phenylglycine t-butyl ester (DAPT, EMD Millipore) or vehicle DMSO 6–8 hours after transfection. For APOE mimetic peptide treatment, transfected HEK293T cells following 24-hours were treated with either 50 μM APOE mimetic peptide (LRVRLASHLRKLRKRLL, Peptide 2.0) or phosphate-buffered saline (PBS) as vehicle control for 30 minutes.

### Biotinylation assay

Media was aspirated 24 hours after transfection, and HEK293T cells were washed with prewarmed PBS. Cells were cooled on ice for 5 minutes and washed once with cold PBS. Cells were incubated with 0.5 mg EZ-link Sulfo-NHS-LC-Biotin (Thermo Fisher Scientific) in cold PBS for 20 minutes at 4°C followed by cold tris-buffered saline (TBS) washes. Cells were lysed with Radioimmunoprecipitation assay (RIPA) buffer (150 mM NaCl, 50 mM Tris-HCl pH 8.0, 1% NP-40, 0.5% sodium deoxycholate, 0.1% SDS, 1 μg/mL leupeptin, 2 μg/mL aprotinin, 1 μg/mL pepstatin), and protein was extracted for 1 hour at 4°C. Samples were centrifuged at 15,000 *g* for 20 minutes at 4°C and supernatant was quantified for protein concentration using Pierce BCA Protein Assay Kit (Thermo Fisher Scientific). Equal amounts of protein were diluted into RIPA buffer as input, and 60 μg protein was incubated with pre-equilibrated Pierce NeutrAvidin Agarose beads (Thermo Fisher Scientific) in RIPA buffer overnight at 4°C. Beads were washed with cold RIPA buffer at 4°C before final resuspension in 2X sample reducing buffer.

### Western blotting

All cell lysates were sonicated for 5 seconds and boiled for 10 minutes at 100°C. All samples were centrifuged at 21,130 *g* for 1 minute. Protein was separated using SDS-PAGE and wet transferred to a nitrocellulose membrane (GE Healthcare). Membranes were blocked for nonspecific binding for 1 hour at room temperature using Odyssey or Intercept Blocking Buffer in PBS (LiCOR-Biosciences). Primary antibodies were diluted into blocking buffer, and membranes were incubated with antibody overnight at 4°C. Primary antibodies were rabbit Apoer2 (1:1000, Abcam ab108208); rabbit Apoer2 against extracellular domain (1:500, 5809, gift from Dr. Joachim Herz’s laboratory); mouse GAPDH (1:5000, Millipore MAB374); mouse tubulin (1:1000, Cell Signaling 3873S); rabbit GFP (1:1000, NeuroMabs 75–131). Secondary antibodies were then diluted 1:20,000 in blocking buffer and incubated for 1 hour at room temperature. All secondary antibodies utilized were raised in goat targeting either human, rabbit, or mouse antibodies and were conjugated to either IRDye680RD or IRDye800CW (Li-COR Biosciences). Final imaging of the membrane was examined using the Odyssey CLX Imaging System (LI-COR Biosciences).

### Primary murine neuronal culture

Primary murine cortical or hippocampal neurons were prepared from individual embryonic day 16.5 *Apoer2* mice (B6;129S6-Lrp8tm1Her/J, stock #003524, The Jackson Laboratory) of either sex that were from a cross of a heterozygous *Apoer2* male with a homozygous *Apoer2* female. Animal studies were approved by the Institutional Animal Care and Use Committee (IACUC). Isolated tissue was washed several times with Hanks Buffered Saline (HBS; Sigma) supplemented to 20% Fetal Select (Atlas Biologicals) (HBS/FBS) followed by HBS before being digested with trypsin and dissociated by trituration, centrifuged at 200 *g* for 15 minutes at 4°C and resuspended in Neuronal Plating Medium [Minimal Essential Medium (MEM, Gibco); 0.033 M glucose (C_6_H_12_O_6_, Sigma); 0.002 M sodium bicarbonate (NaHCO_3_, Sigma); 0.1 mg/mL transferrin (Calbiochem); 10% Fetal Select (Atlas Biologicals); 2 mM L-glutamine (Gibco); 0.025 mg/mL insulin (Sigma)]. Neurons were plated onto poly-L-lysine (Corning) coated wells or 12 mm glass coverslips (Carolina Biological Supply) and maintained at 37°C with 5% CO_2_. The day after plating, neurons were switched into Neuronal Growth Medium [Neurobasal Media (Gibco); 0.5 mM L-glutamine (Gibco); 1% B-27 Supplement (Gibco)].

### Lentivirus generation and testing

HEK293T cells were transfected with viral packaging plasmids (VSVG, REV and RRE) and a shuttle vector carrying the APOER2 plasmid of interest using FuGene6 (Roche). After 16 hours, media was replaced with Neuronal Growth Media. 48 hours after transfection, supernatant containing generated lentiviral particles was collected and centrifuged at 500 *g* for 5 minutes at 4°C. Supernatant was filtered using a 0.45 μM filter (GE Healthcare), aliquoted and stored at -80°C until use. For lentiviral infection of primary neurons, lentivirus was added directly to media on 1 day *in vitro* (DIV) with lentivirus. An infection curve was performed to ensure equal infection across all *APOER2* isoforms (S5 Fig).

### Immunocytochemistry and image analysis

Neurons were fixed in 4% paraformaldehyde (Thermo Fisher Scientific), blocked in 10% goat serum and permeabilized with 0.1% saponin in PBS followed by incubation with primary antibodies in blocking buffer (10% goat serum in PBS) at 4°C overnight. The primary antibodies used included mouse PSD-95 (1:200, Novus Biologicals NB300-556) and rabbit synapsin (1:500, P610, gift from Dr. Thomas Südhof’s laboratory). Following PBS washes, neurons were incubated with fluorophore-conjugated secondary antibodies: goat anti-rabbit IgG Alexa Fluor 488 (1:500, Invitrogen) and goat anti-mouse IgG Alexa Fluor 546 (1:500, Invitrogen). Coverslips were mounted on SuperFrost microscope slides (Fisher Scientific) in ProLong-Gold Anti-fade mount with DAPI (Invitrogen).

Images were captured using a Carl Zeiss LSM700 scanning confocal microscope. Image acquisition settings were kept constant between coverslips and independent experiments, including settings for the laser gain and offset, scanning speed, and pinhole size. 3D Z-stacks were acquired of the neuronal processes using a 63x oil objective. The region of interest was selected manually on each image using well-isolated primary dendrites. 3D images were analyzed in Imaris (Oxford Instruments) for synapse number and area using the Surface-Surface Colocalization XTension which runs a MATLAB algorithm. In short, all images were segmented into regions of interest of a uniform size which encompassed isolated neuronal processes. 3D surfaces were generated for the synapsin and PSD-95 channel with a 10 voxel drop off. Threshold and surface detail values were kept consistent for each channel across experiments. A colocalized surface was then generated using the synapsin and PSD-95 surfaces based on their area of overlap.

### Statistical analysis

All statistical analysis was performed as described in figure legends using GraphPad Prism v9 with α = 0.05. Statistical significance was determined using either a Student’s *t*-test to compare two groups, or a one-way ANOVA with posthoc Dunnett’s or Tukey’s multiple comparisons test. All the numerical data that underlies graphs and summary statistics are listed in supporting information **S1 Data**.

## Supporting information

S1 Fig Cumulative frequency plots for parietal cortex and hippocampus. (A) Graph depicting the cumulative frequency of detected isoforms in each of the six parietal cortex samples. (B) Graph depicting the cumulative frequency of detected isoforms in each of the six hippocampal samples.
(TIF)

S2 Fig Pairwise scatterplots comparing APOER2 transcripts within brain region and across brain region in control and AD. (A-B) Scatterplots of the ranked median *APOER2* TPM for the (A) parietal cortex or (B) hippocampus AD versus control samples. Blue indicates isoforms common between the parietal cortex and hippocampus, while grey indicates a transcript only identified in that region. Transcript numbering is comparable between A & B.
(TIF)

S3 Fig Traceback of APOER2 isoform PB.79.480 in sample AD#1. Isoform sequences associated with PB.79.480 in the hippocampus at different stages of analysis, including the high-quality (hq) IsoSeq output, post-cupcake collapse and post-SQANTI3 reference correction sequence, were aligned with the *APOER2* human NCBI reference sequence for analysis of exon composition. Sequence indicated exclusion of ex5 and addition of highlighted (red) 11 bases before ex18, that correspond to intronic sequence just before ex18 and do not disturb the open reading frame as shown by amino acids added in red. Alignment was performed with SnapGene 6.0 software.
(TIF)

S4 Fig Amplicon sequence validating RT-PCR primers targeting APOER2 ex15. Sanger sequencing indicated inclusion of *APOER2* ex15 aligned with the *APOER2* human NCBI reference sequence for analysis of exon composition.
(TIF)

S5 Fig Expression of lentiviral APOER2 variants in primary murine neurons. Representative immunoblots showing neuronal lysates from wildtype murine neurons that were infected with human lentiviral GFP-tagged APOER2 variants with increasing % of lentivirus (0.5, 1, 2.5), V = pFUW only (lane 8), and uninfected (lane 9) using anti-APOER2 C-terminal and GFP antibodies. GAPDH served as loading ntrol.
(TIF)

S1 Table Individual sample full-length (FL) read statistics. (DOCX)

S2 Table APOER2 isoforms unique to either control or AD in the parietal cortex. (DOCX)

S3 Table Exons annotated in APOER2 transcripts across parietal cortex and hippocampus long-read sequencing experiments. (DOCX)

S4 Table APOER2 exons annotated in GTEx database compared to identified exons in long-read sequencing experiments. (DOCX)

S5 Table APOER2 isoforms unique to either control or AD in the hippocampus. (DOCX)

S6 Table APOER2 isoforms in the parietal cortex and hippocampus that were in the top 10 isoforms in one region, but not the other. (DOCX)

S7 Table BioSample and SRA accession numbers for long-read RNAseq datasets. (DOCX)

S1 Data All the numerical data that underlies graphs and summary statistics. (XLSX)
