# Supplementary figures and images for: APOER2 splicing repertoire in Alzheimer’s disease: Insights from long-read RNA sequencing
Source: PLoS Genet. 2024 Jul 22;20(7):e1011348. doi: 10.1371/journal.pgen.1011348 (PMC11293713; doi:10.1371/journal.pgen.1011348)

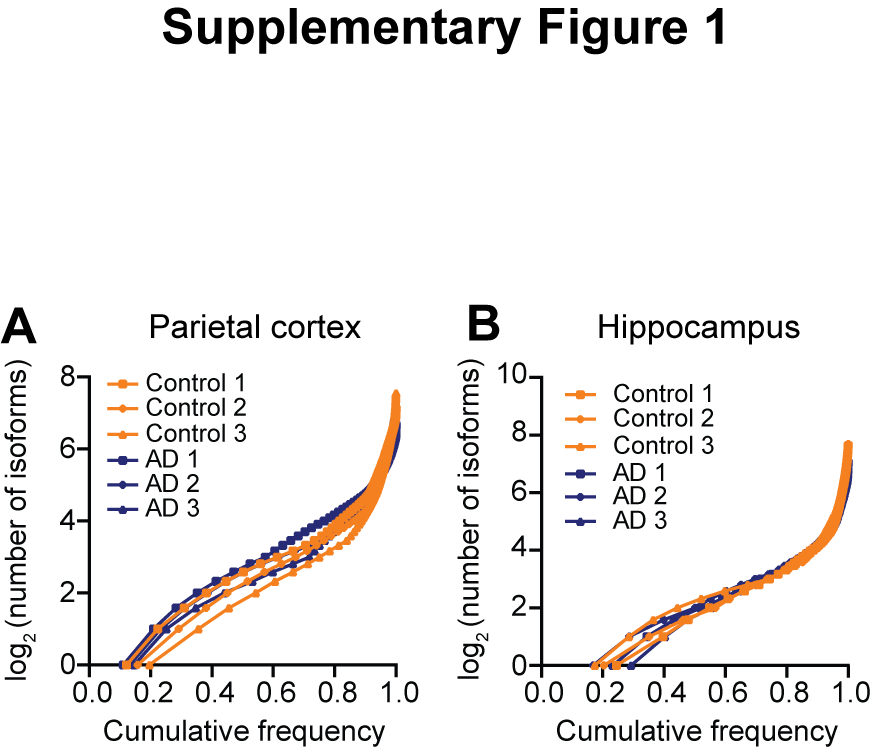

Supplement: S1 Fig — (A) Graph depicting the cumulative frequency of detected isoforms in each of the six parietal cortex samples. (B) Graph depicting the cumulative frequency of detected isoforms in each of the six hippocampal samples. (TIF) [file pgen.1011348.s001.tif]

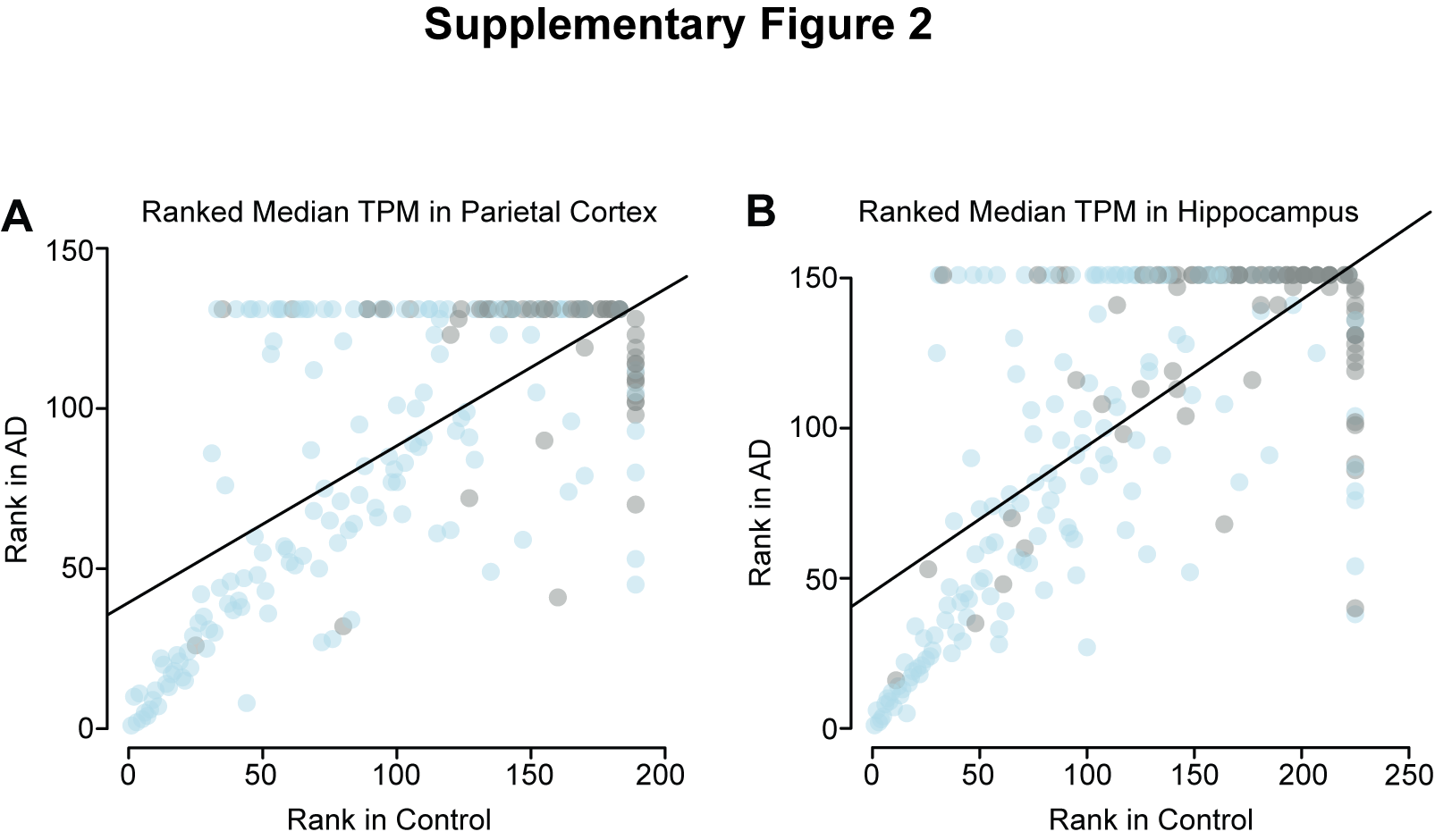

Supplement: S2 Fig — (A-B) Scatterplots of the ranked median APOER2 TPM for the (A) parietal cortex or (B) hippocampus AD versus control samples. Blue indicates isoforms common between the parietal cortex and hippocampus, while grey indicates a transcript only identified in that region. Transcript numbering is comparable between A & B. (TIF) [file pgen.1011348.s002.tif]

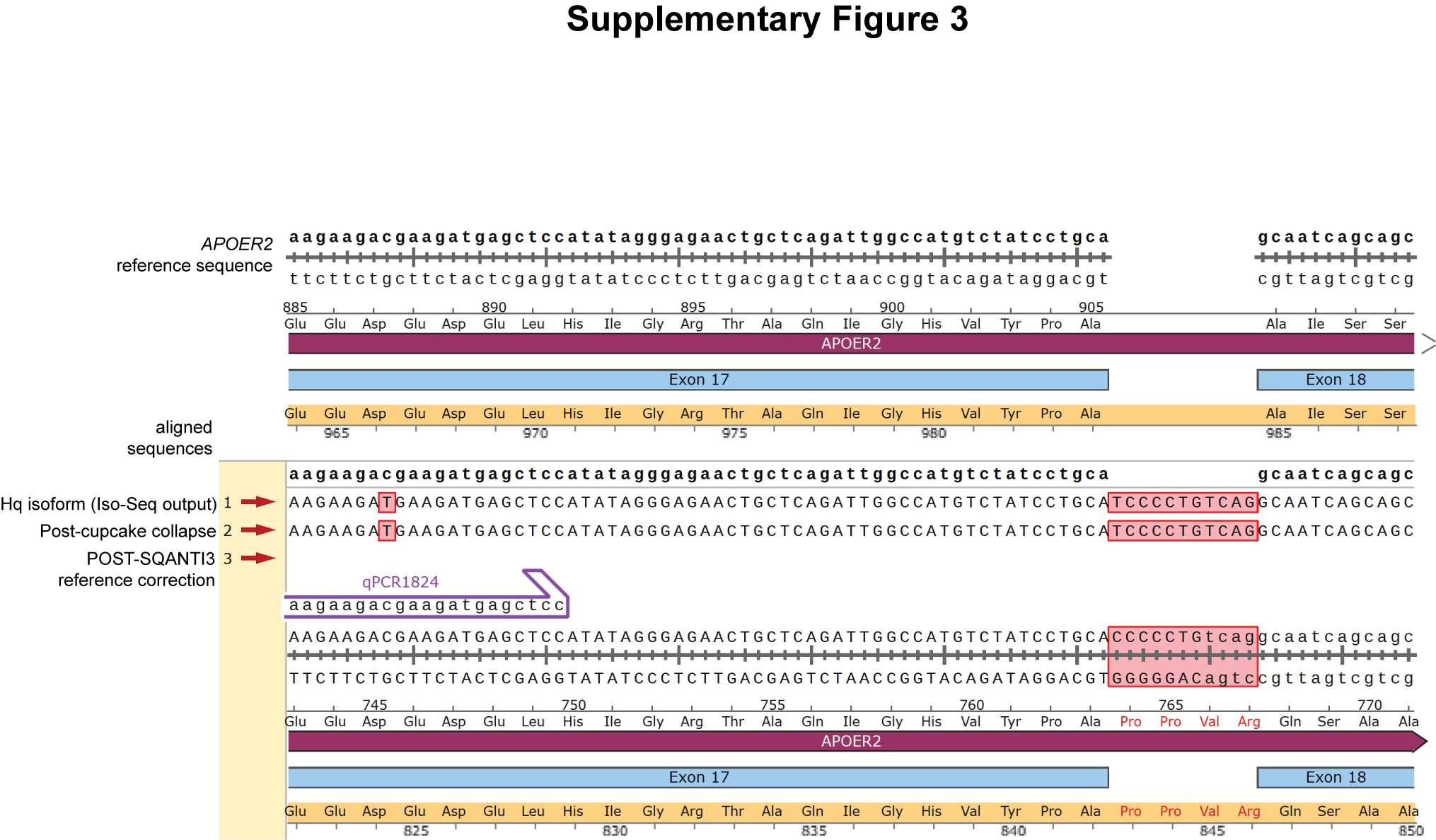

Supplement: S3 Fig — Isoform sequences associated with PB.79.480 in the hippocampus at different stages of analysis, including the high-quality (hq) IsoSeq output, post-cupcake collapse and post-SQANTI3 reference correction sequence, were aligned with the APOER2 human NCBI reference sequence for analysis of exon composition. Sequence indicated exclusion of ex5 and addition of highlighted (red) 11 bases before ex18, that correspond to intronic sequence just before ex18 and do not disturb the open reading frame as shown by amino acids added in red. Alignment was performed with SnapGene 6.0 software. (TIF) [file pgen.1011348.s003.tif]

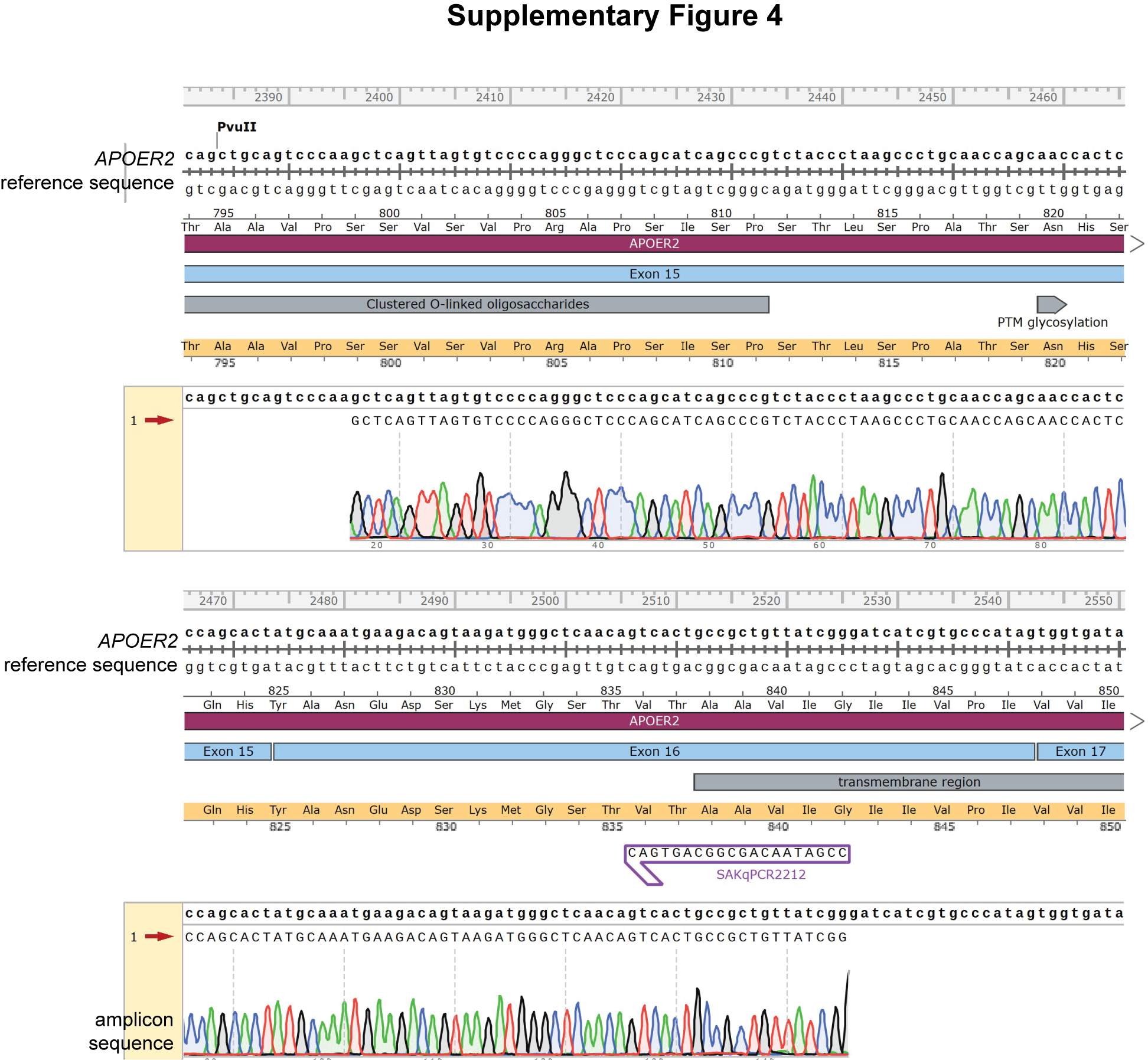

Supplement: S4 Fig — Sanger sequencing indicated inclusion of APOER2 ex15 aligned with the APOER2 human NCBI reference sequence for analysis of exon composition. (TIF) [file pgen.1011348.s004.tif]

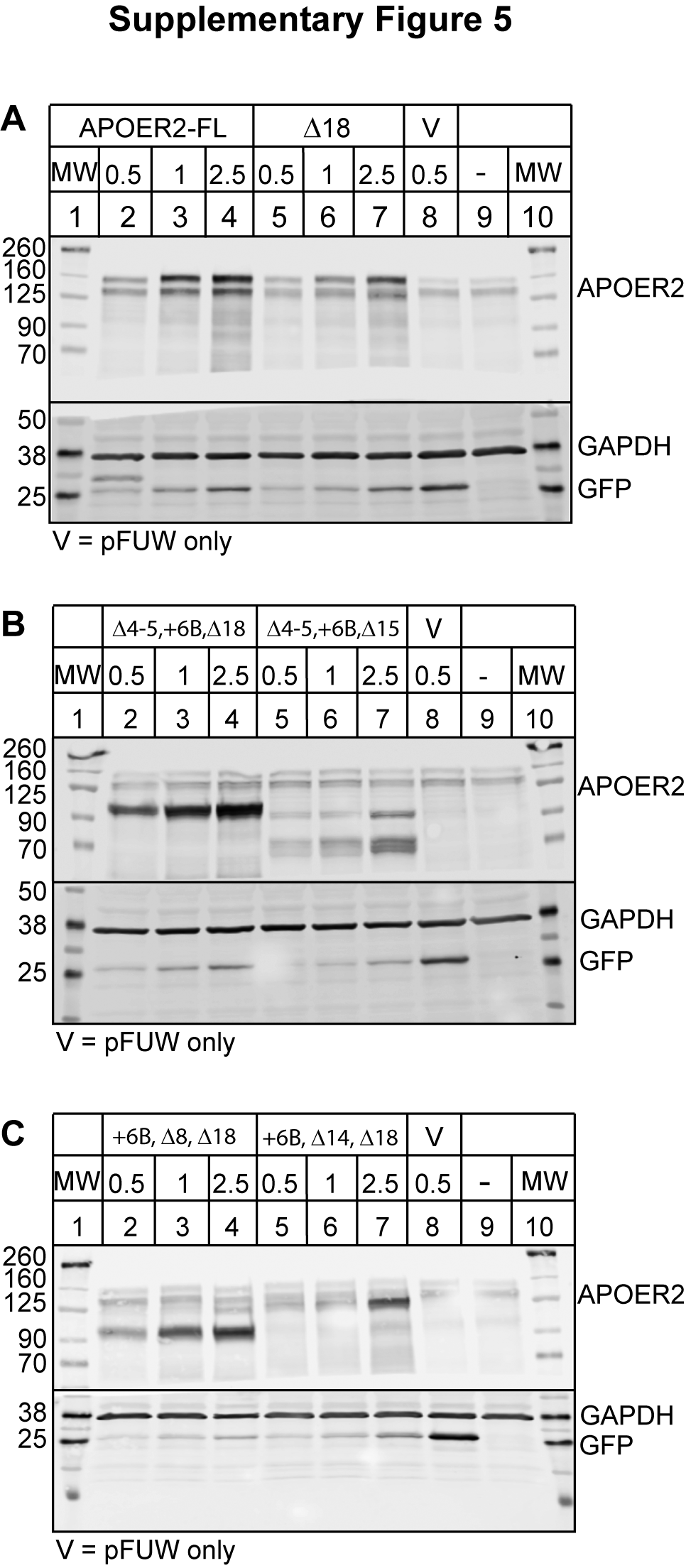

Supplement: S5 Fig — Representative immunoblots showing neuronal lysates from wildtype murine neurons that were infected with human lentiviral GFP-tagged APOER2 variants with increasing % of lentivirus (0.5, 1, 2.5), V = pFUW only (lane 8), and uninfected (lane 9) using anti-APOER2 C-terminal and GFP antibodies. GAPDH served as loading ntrol. (TIF) [file pgen.1011348.s005.tif]
