# Supplementary material for: APOER2 splicing repertoire in Alzheimer’s disease: Insights from long-read RNA sequencing
Source: PLoS Genet. 2024 Jul 22;20(7):e1011348. doi: 10.1371/journal.pgen.1011348 (PMC11293713; doi:10.1371/journal.pgen.1011348)
Supplement: S1 Table — (DOCX) [file pgen.1011348.s006.docx]

**S1 Table: Individual sample full-length (FL) read statistics**

| **Brain Region** | **Sample** | **Total FL Reads** | **# *APOER2* FL Reads** | **% *APOER2* FL reads** |
| --- | --- | --- | --- | --- |
| Parietal cortex | AD 1 | 148666 | 108993 | 73% |
| Parietal cortex | AD 2 | 148035 | 134837 | 91% |
| Parietal cortex | AD 3 | 149735 | 112792 | 75% |
| Parietal cortex | Control 1 | 147791 | 124674 | 84% |
| Parietal cortex | Control 2 | 146934 | 133629 | 91% |
| Parietal cortex | Control 3 | 147762 | 134115 | 91% |
| Hippocampus | AD 1 | 196260 | 173665 | 88% |
| Hippocampus | AD 2 | 216716 | 189769 | 88% |
| Hippocampus | AD 3 | 99755 | 72338 | 73% |
| Hippocampus | Control 1 | 142833 | 123762 | 87% |
| Hippocampus | Control 2 | 179653 | 158941 | 88% |
| Hippocampus | Control 3 | 125963 | 102907 | 82% |
