# Supplementary material for: APOER2 splicing repertoire in Alzheimer’s disease: Insights from long-read RNA sequencing
Source: PLoS Genet. 2024 Jul 22;20(7):e1011348. doi: 10.1371/journal.pgen.1011348 (PMC11293713; doi:10.1371/journal.pgen.1011348)
Supplement: S2 Table — (DOCX) [file pgen.1011348.s007.docx]

**S2 Table: *APOER2* isoforms unique to either control or AD in the parietal cortex**

| **Group** | **Isoform** | **Exon Annotation** |
| --- | --- | --- |
| Control | PB.97.1158 | +ex6B, Δex8, Δex15, Δex18 |
| Control | PB.97.1196 | Δex5-6, +ex6B, Δex18 |
| Control | PB.97.941 | Δex5-6, Δex8, Δex15, Δex18 |
| Control | PB.97.326 | Retained intron between ex7-8, Δex15 |
| Control | PB.97.1145 | Δex5-6, +c.ex. between ex14-15, Δex18 |
| Control | PB.97.387 | Δex10 |
| Control | PB.97.311 | +ex6B, Δex14-16 |
| Control | PB.97.1104 | Δex5, Δex10, Δex18 |
| Control | PB.97.1010 | Δex5, a5’ss in ex8, Δex14-15, Δex18 |
| Control | PB.97.1093 | Ex7-retained intron-ex8, Δex15, Δex18 |
| Control | PB.97.807 | c.ex.#2, +ex6B |
| Control | PB.97.1005 | Δex14-16, Δex18 |
| Control | PB.97.481 | Δex5, +ex6B, a5’ss in ex8, Δex15 |
| Control | PB.97.462 | Δex5-6, +ex6B |
| Control | PB.97.1491 | Δex5, Δex16-18 |
| Control | PB.97.761 | Δex5, c.ex.#1, c.ex. between ex14-15 |
| Control | PB.97.275 | Δex5, Δex10 |
| Control | PB.97.382 | +ex6B, Δex10, Δex15 |
| Control | PB.97.427 | c.ex.#1, Δex8, Δex15 |
| Control | PB.97.1148 | Δex5, +ex6B, Δex14, Δex18 |
| AD | PB.97.979 | Δex4-5, +ex6B, Δex14-15, Δex18 |
| AD | PB.97.138 | Δex5-6, ex7-retained intron-ex8 |
| AD | PB.97.136 | Δex4-5, +ex6B, Δex14-15 |
| AD | PB.97.918 | Δex5-6, ex7-retained intron-ex8, Δex15, Δex18 |
| AD | PB.97.1013 | Δex5, +ex6B, Δex8, Δex14-15, Δex18 |
| AD | PB.97.147 | Δex5, Δex11, Δex15 |
