# Supplementary material for: APOER2 splicing repertoire in Alzheimer’s disease: Insights from long-read RNA sequencing
Source: PLoS Genet. 2024 Jul 22;20(7):e1011348. doi: 10.1371/journal.pgen.1011348 (PMC11293713; doi:10.1371/journal.pgen.1011348)
Supplement: S3 Table — (DOCX) [file pgen.1011348.s008.docx]

**S3 Table: Exons annotated in *APOER2* transcripts across parietal cortex and hippocampus long-read sequencing experiments.**

| **Exon Annotation** | **Start Genomic Coordinate** | **End Genomic Coordinate** | **Present in Parietal Cortex?** | **Present in Hippocampus?** | **Ensembl Exon** |
| --- | --- | --- | --- | --- | --- |
| a3’ss in ex18 | 53249380 | 53249440 | Y | Y | ENSE00003867106 |
| ex18 | 53249380 | 53249556 | Y | Y | ENSE00003513209 |
| ex17 | 53250690 | 53250862 | Y | Y | ENSE00003524622 |
| ex16 | 53255117 | 53255185 | Y | Y | ENSE00003594882 |
| ex15 | 53257240 | 53257464 | Y | Y | ENSE00003523443 |
| c.ex between ex14-15 | 53258009 | 53258074 | Y | Y | ENSE00001900254 |
| ex14 | 53258319 | 53258471 | Y | Y | ENSE00003642659 |
| ex13 | 53260464 | 53260605 | Y | Y | ENSE00003568292 |
| ex12 | 53262068 | 53262207 | Y | Y | ENSE00003489729 |
| ex11-retained intron-ex12 | 53262068 | 53262564 | N | Y | N/A |
| ex11 | 53262446 | 53262564 | Y | Y | ENSE00003510050 |
| ex10 | 53264169 | 53264396 | Y | Y | ENSE00003784065 |
| ex9 | 53266473 | 53266647 | Y | Y | ENSE00003609939 |
| ex8 | 53271028 | 53271153 | Y | Y | ENSE00003461529 |
| ex7-retained intron-ex8 | 53271028 | 53271346 | Y | Y | N/A |
| a5’ss in ex8 | 53271085 | 53271153 | Y | Y |  |
| ex7 | 53271227 | 53271346 | Y | Y | ENSE00003500091 |
| a3’ss ex7 | 53271227 | 53271415 |  | Y | ENSE00003498719 |
| ex6B | 53272604 | 53272642 | Y | Y | ENSE00001946612 |
| c.ex.#1 | 53274483 | 53274820 | Y | Y |  |
| c.ex.#2 | 53274643 | 53274820 | Y | N |  |
| ex6 | 53275631 | 53275753 | Y | Y | ENSE00003632064 |
| ex5 | 53276692 | 53277078 | Y | Y | ENSE00001031081 |
| ex4 | 53280587 | 53280715 | Y | Y | ENSE00003489712 |
| ex3 | 53289567 | 53289689 | Y | Y | ENSE00003664024 |
| ex2 | 53326873 | 53326992 | Y | Y | ENSE00003585859 |
| ex1 | 53327789 | 53327895 | Y | Y | Part of ENSE00003879526 |
