# Supplementary material for: APOER2 splicing repertoire in Alzheimer’s disease: Insights from long-read RNA sequencing
Source: PLoS Genet. 2024 Jul 22;20(7):e1011348. doi: 10.1371/journal.pgen.1011348 (PMC11293713; doi:10.1371/journal.pgen.1011348)
Supplement: S4 Table — (DOCX) [file pgen.1011348.s009.docx]

**S4 Table: *APOER2* exons annotated in GTEx database compared to identified exons in long-read sequencing experiments**

Genomic coordinates shared are highlighted in yellow, and exons not present are shaded in grey.

| **GTEx Exon** | **Start** | **End** | **PacBio exon annotation** | **Start** | **End** | **Present in both regions?** |
| --- | --- | --- | --- | --- | --- | --- |
| 1 | 53327789 | 53328070 | ex1 | 53327789 | 53327895 | Y |
| 2 | 53327258 | 53327399 |  |  |  |  |
| 3 | 53326873 | 53326992 | ex2 | 53326873 | 53326992 | Y |
| Exon without # | 53289567 | 53289689 | ex3 | 53289567 | 53289689 | Y |
| 4 | 53280587 | 53280715 | ex4 | 53280587 | 53280715 | Y |
| 5 | 53276692 | 53277078 | ex5 | 53276692 | 53277078 | Y |
| 6 | 53275631 | 53275753 | ex6 | 53275631 | 53275753 | Y |
| 7 | 53274700 | 53274820 |  |  |  |  |
|  |  |  | c.ex. #2 | 53274643 | 53274820 | N- PARCTX only |
|  |  |  | c.ex. #1 | 53274483 | 53274820 | Y |
| 8 | 53272604 | 53272642 | ex6B | 53272604 | 53272642 | Y |
|  |  |  | a3’ss ex7 | 53271227 | 53271415 | N- HIPP only |
| 9 | 53271227 | 53271346 | ex7 | 53271227 | 53271346 | Y |
|  |  |  | a5’ss in ex8 | 53271085 | 53271153 | Y |
|  |  |  | ex7-intron-ex8 | 53271028 | 53271346 | Y |
| 10 | 53271028 | 53271153 | ex8 | 53271028 | 53271153 | Y |
| 11 | 53266473 | 53266647 | ex9 | 53266473 | 53266647 | Y |
| 12 | 53264169 | 53264396 | ex10 | 53264169 | 53264396 | Y |
| 13 | 53262446 | 53262564 | ex11 | 53262446 | 53262564 | Y |
|  |  |  | ex11-intron-ex12 | 53262068 | 53262564 | N- HIPP only |
| 14 | 53262068 | 53262207 | ex12 | 53262068 | 53262207 | Y |
| 15 | 53260464 | 53260605 | ex13 | 53260464 | 53260605 | Y |
| 16 | 53258319 | 53258471 | ex14 | 53258319 | 53258471 | Y |
| 17 | 53258009 | 53258074 | c.ex between ex14-15 | 53258009 | 53258074 | Y |
| 18 | 53257240 | 53257464 | ex15 | 53257240 | 53257464 | Y |
| 19 | 53255117 | 53255185 | ex16 | 53255117 | 53255185 | Y |
| 20 | 53250690 | 53250862 | ex17 | 53250690 | 53250862 | Y |
| 21 | 53249380 | 53249556 | ex18 | 53249380 | 53249556 | Y |
|  |  |  | a3’ss in ex18 | 53249380 | 53249440 | Y |
| 22 | 53242784 | 53247056 |  |  |  |  |
| 23 | 53242364 | 53242437 |  |  |  |  |
