# Supplementary material for: APOER2 splicing repertoire in Alzheimer’s disease: Insights from long-read RNA sequencing
Source: PLoS Genet. 2024 Jul 22;20(7):e1011348. doi: 10.1371/journal.pgen.1011348 (PMC11293713; doi:10.1371/journal.pgen.1011348)
Supplement: S5 Table — (DOCX) [file pgen.1011348.s010.docx]

**S5 Table: *APOER2* isoforms unique to either control or AD in the hippocampus**

| **Group** | **Isoform** | **Exon Annotation** |
| --- | --- | --- |
| Control | PB.79.1318 | +ex6B, Δex8, Δex18 |
| Control | PB.79.215 | ex7- retained intron-ex8, Δex15 |
| Control | PB.79.636 | +ex6B, a5’ss in ex8, Δex15 |
| Control | PB.79.394 | +ex6B, Δex7, Δex15 |
| Control | PB.79.1100 | Δex5, Δex10, Δex18 |
| Control | PB.79.241 | Δex5, Δex11 |
| Control | PB.79.1433 | +ex6B, a3’ss ex7, Δex18 |
| Control | PB.79.1340 | Δex5, +ex6B, a3’ss ex7, Δex18 |
| Control | PB.79.560 | Δex5, +ex6B, ex7-retained intron-ex8, c.ex. between ex14-15 |
| Control | PB.79.134 | Δex5, ex11-retained intron-ex12, Δex15 |
| Control | PB.79.542 | +ex6B, Δex8, a3’ss in ex18 |
| Control | PB.79.1126 | Δex5, +ex6B, ex7-retained intron-ex8, Δex15, Δex18 |
| Control | PB.79.794 | +ex6B, a3’ss ex7 |
| Control | PB.79.661 | Δex5, +ex6B, a3’ss ex7 |
| Control | PB.79.118 | Δex5, Δex8, Δex14 |
| Control | PB.79.1250 | a3’ss ex7, Δex15, Δex18 |
| Control | PB.79.1082 | Δex10, Δex15, Δex18 |
| Control | PB.79.88 | Δex4-5, Δex14-15 |
| Control | PB.79.1016 | Δex5, Δex11, Δex15, Δex18 |
| Control | PB.79.1336 | Δex5, +ex6B, Δex7, +c.ex. between ex14-15, Δex18 |
| Control | PB.79.1591 | Δex5, Δex14-18 |
| Control | PB.79.289 | Δex5, a3’ss ex7, Δex8 |
| Control | PB.79.1037 | Δex5, ex7-retained intron-ex8, Δex11, Δex18 |
| Control | PB.79.1149 | Δex5, +ex6B, Δex10, Δex15, Δex18 |
| Control | PB.79.300 | a3’ss ex7, Δex14-15 |
| Control | PB.79.391 | Δex5, +ex6B, a5’ss in ex8, Δex15 |
| Control | PB.79.119 | Δex5-6, a5’ss in ex8, Δex15 |
| Control | PB.79.1029 | Δex5, Δex10, Δex15, Δex18 |
| Control | PB.79.1454 | Δex4, +ex6B, c.ex. between ex14-15, Δex18 |
| Control | PB.79.438 | Δex5, ex11-retained intron-ex12, c.ex. between ex14-15 |
| Control | PB.79.400 | c.ex.#1, Δex8, Δex15 |
| Control | PB.79.491 | Δex5, +ex6B, Δex14 |
| Control | PB.79.992 | Δex4-5, +ex6B, Δex14-15, Δex18 |
| Control | PB.79.77 | Δex4-6, Δex10 |
| Control | PB.79.1599 | Δex10-18 |
| Control | PB.79.430 | Δex5, c.ex.#1, Δex15 |
| Control | PB.79.1371 | +ex6B, ex11-retained intron-ex12, Δex18 |
| AD | PB.79.217 | Δex5, +ex6B, ex11-retained intron-ex12, Δex15 |
| AD | PB.79.985 | Δex5, a3’ss ex7, Δex8, Δex15, Δex18 |
| AD | PB.79.1350 | Δex4-5, +ex6B, c.ex. between ex14-15, Δex18 |
| AD | PB.79.169 | Δex5, Δex8, Δex15, a3’ss in ex18 |
| AD | PB.79.1291 | +ex6B, Δex14, Δex18 |
