# Supplementary material for: APOER2 splicing repertoire in Alzheimer’s disease: Insights from long-read RNA sequencing
Source: PLoS Genet. 2024 Jul 22;20(7):e1011348. doi: 10.1371/journal.pgen.1011348 (PMC11293713; doi:10.1371/journal.pgen.1011348)
Supplement: S6 Table — (DOCX) [file pgen.1011348.s011.docx]

**S6 Table: *APOER2* isoforms in the parietal cortex and hippocampus that were in the top 10 isoforms in one region, but not the other**

Coloring indicates which region contained the isoform within the top 10. Parietal cortex (Par ctx), hippocampus (Hipp)

| **Region** | **Isoform** | **Exon Annotation** | **TPM**  **Control1** | **TPM Control2** | **TPM Control3** | **TPM AD1** | **TPM**  **AD2** | **TPM AD3** | **padj** |
| --- | --- | --- | --- | --- | --- | --- | --- | --- | --- |
| Par ctx | PB.97.231 | Δex4-5, +ex6B, Δex15 | 15716 | 782 | 1124 | 50267 | 1372 | 53407 | 0.879 |
| Hipp | PB.79.192 | Δex4-5, +ex6B, Δex15 | 0 | 529 | 0 | 259 | 0 | 660 | 0.99 |
| Par ctx | PB.97.1038 | Δex5, Δex8, Δex18 | 12518 | 16692 | 8240 | 22126 | 14470 | 16592 | 0.998 |
| Hipp | PB.79.1060 | Δex5, Δex8, Δex18 | 7948 | 5131 | 4164 | 16 | 7157 | 6644 | 0.99 |
| Par ctx | N/A | Δex5, Δex15 |  |  |  |  |  |  |  |
| Hipp | PB.79.189 | Δex5, Δex15 | 16194 | 46335 | 18518 | 10669 | 14544 | 23165 | 0.99 |
| Par ctx | PB.97.154 | Δex4-6 | 1312 | 694 | 1392 | 3873 | 1476 | 0 | 0.998 |
| Hipp | PB.79.103 | Δex4-6 | 5372 | 8744 | 15064 | 70904 | 8536 | 75612 | 0.783 |
| Par ctx | PB.97.1250 | +ex6B, Δex15, Δex18 | 23809 | 18695 | 4552 | 9868 | 20326 | 3235 | 0.918 |
| Hipp | PB.79.1292 | +ex6B, Δex15, Δex18 | 15254 | 5380 | 12998 | 28479 | 19555 | 8145 | 0.99 |
