# Supplementary material for: APOER2 splicing repertoire in Alzheimer’s disease: Insights from long-read RNA sequencing
Source: PLoS Genet. 2024 Jul 22;20(7):e1011348. doi: 10.1371/journal.pgen.1011348 (PMC11293713; doi:10.1371/journal.pgen.1011348)
Supplement: S7 Table — (DOCX) [file pgen.1011348.s012.docx]

**S7 Table: BioSample and SRA accession numbers for long-read RNAseq datasets**

| **Sample Name** | **BioSample Accession** | **SRA Accession** | **BioSample URL** |
| --- | --- | --- | --- |
| Control_1_HC | SAMN41494590 | SRR29131174 | https://www.ncbi.nlm.nih.gov/sra/41494590 |
| Control_2_HC | SAMN41494591 | SRR29131173 | https://www.ncbi.nlm.nih.gov/sra/41494591 |
| Control_3_HC | SAMN41494592 | SRR29131170 | https://www.ncbi.nlm.nih.gov/sra/41494592 |
| Control_1_PC | SAMN41494593 | SRR29131166 | https://www.ncbi.nlm.nih.gov/sra/41494593 |
| Control_2_PC | SAMN41494594 | SRR29131165 | https://www.ncbi.nlm.nih.gov/sra/41494594 |
| Control_3_PC | SAMN41494595 | SRR29131164 | https://www.ncbi.nlm.nih.gov/sra/41494595 |
| AD_1_HC | SAMN41494596 | SRR29131169 | https://www.ncbi.nlm.nih.gov/sra/41494596 |
| AD_2_HC | SAMN41494597 | SRR29131168 | https://www.ncbi.nlm.nih.gov/sra/41494597 |
| AD_3_HC | SAMN41494598 | SRR29131167 | https://www.ncbi.nlm.nih.gov/sra/41494598 |
| AD_1_PC | SAMN41494599 | SRR29131163 | https://www.ncbi.nlm.nih.gov/sra/41494599 |
| AD_2_PC | SAMN41494600 | SRR29131172 | https://www.ncbi.nlm.nih.gov/sra/41494600 |
| AD_3_PC | SAMN41494601 | SRR29131171 | https://www.ncbi.nlm.nih.gov/sra/41494601 |

BioProject SRA ID: PRJNA1114762
